# Toward Holistic Aging in Lebanon: A Mapping of Gerontology and Geriatrics Research Trends, Gaps, and Future Directions

**DOI:** 10.3390/healthcare14172857

**Published:** 2026-09-04

**Authors:** Rudy S. Younes, Charbel Ziade, Ferial Rajha, Mirna Abboud Mzawak

**Affiliations:** 1 Department of Psychology and Social Sciences, Faculty of Arts and Sciences, Holy Spirit University of Kaslik (USEK), Jounieh P.O. Box 446, Keserwan, Lebanon; rudy.younes@usek.edu.lb (R.S.Y.); charbel.e.ziade@net.usek.edu.lb (C.Z.); 2 IDEES Multidisciplinary Research Group, Faculty of Arts and Sciences, Holy Spirit University of Kaslik (USEK), Jounieh P.O. Box 446, Keserwan, Lebanon; 3 Faculty of Public Health, Lebanese University, Badaro P.O. Box 6573/14, Beirut, Lebanon; ferial.rajha@ul.edu.lb; 4 Department of Nursing, Faculty of Public Health, Lebanese German University (LGU), Jounieh P.O. Box 206, Keserwan, Lebanon

**Keywords:** holistic aging, gerontology, geriatrics, Lebanon, psychosocial well-being, social gerontology, community gerontology, aging in place

## Abstract

**Background/Objectives:** Lebanon is undergoing a rapid demographic transition, with older adults projected to exceed one-fifth of the population by 2050. Supporting healthy and independent aging requires a robust evidence base that extends beyond clinical care to encompass community, environmental, psychosocial, and policy dimensions of aging in place. This bibliometric review maps the landscape of gerontology and geriatrics research in Lebanon to identify research trends, gaps, and future priorities for advancing holistic approaches to aging. **Methods:** A bibliometric review was conducted of gerontology and geriatrics research published between 2015 and 2026. Publications were retrieved from the Scopus database and screened according to predefined eligibility criteria. **Results:** A total of 403 publications met the inclusion criteria. Research productivity increased steadily over the study period, reaching its highest level in 2025, although publications remained concentrated among a relatively small number of authors and institutions. The literature was predominantly oriented toward clinical, epidemiological, and public health aspects of aging, with comparatively limited attention to community-based aging, psychosocial well-being, age-friendly environments, policy, social participation, and other determinants that support older adults’ ability to age safely and independently within their communities. Research evaluating interventions outside tertiary healthcare settings was particularly scarce. **Conclusions:** This review provides the first bibliometric mapping of gerontology and geriatrics research in Lebanon and indicates that, despite substantial growth in scientific output, the identified literature is largely centered on diseases and institutional healthcare. Future research should adopt more holistic and multidisciplinary perspectives to promote psychosocial well-being and aging well in one’s community. These priorities may guide researchers, practitioners, and policymakers in developing strategies for aging well in Lebanon.

## 1. Introduction

The world population is undergoing a significant demographic shift, with 1 in 9 individuals aged 60 or older estimated to reach 1 in 5 by 2050 [1]. With the increase in the elderly population, the need for better healthcare services and long-term care is crucial [2]. Due to unprecedented population aging, gerontology research has surged worldwide [3,4], providing important advances in understanding aging experiences and developing interventions.

This aging trend is also clear in the Middle East, namely in Lebanon. In fact, the country has the fastest-growing older adult population in the region. As early as 1995, 5.4% of the Lebanese population was over 65, rising to 8% by 2001 [5,6]. By 2012, Lebanon reported the highest percentage of adults aged 65+, with a proportion of 10% [7,8]. This percentage is expected to be more than double, approaching 21% by the year 2050 [9]. However, Lebanon currently lacks the required resources to address the needs of this growing demographic [10]. This lack of resources is evidenced by insufficient healthcare staffing, financial barriers, and over half of Lebanese older adults lacking healthcare insurance in 2015 [11,12].

The older adult population faces a range of unique challenges. For instance, the literature has consistently shown that older adults are affected by a variety of health conditions that become more prevalent with age [13]. Frailty, for example, is a common condition characterized by physical weakness and an increased vulnerability to falls, hospitalization, and functional decline [14]. Several non-communicable diseases are also more prevalent among older adults and may occur simultaneously, resulting in multimorbidity [15]. Moreover, functional decline and non-communicable diseases represent major contributors to disability and reduced quality of life in later years [16]. Finally, mental health problems constitute another important concern, as older adults are particularly susceptible to the underdiagnosis and undertreatment of mental health conditions, with depression, anxiety, and stress among the most prevalent challenges in later life [17].

In Lebanon, late adulthood seems to be further complicated by the country’s distinct sociocultural characteristics. Indeed, the country has experienced socioeconomic instability for the past couple of decades, as well as recurrent episodes of wars and armed conflicts, which have led to a fragile healthcare system [18]. More recently, Lebanon has faced a series of overlapping crises, including a severe economic collapse beginning in 2019, the COVID-19 pandemic, the Beirut Port Explosion, one of the largest non-nuclear explosions, in 2020, and armed conflict since 2023. Together, these events have led to huge pressure on healthcare and social support systems, and have increased the needs of vulnerable populations, including older adults [19].

Most importantly, there are considerable barriers that complicate healthcare access and services for older Lebanese individuals. Some of these are staff shortages, bad service organization, and financial challenges, among other factors [11]. Moreover, according to Osta et al. [12], 55% of the Lebanese elderly in 2015 had no health insurance, further exacerbating the challenges experienced by older adults in Lebanon. In light of these major institutional challenges, community health services and community gerontology can be seen as valuable ways of supporting older adults in Lebanon [20]. In fact, communities represent the immediate physical, social, and structural contexts within which aging occurs. Community-based networks, local non-governmental organizations, and informal support systems can be essential mechanisms that enable older adults to age in their own homes or communities, and with dignity [21].

Importantly, the challenges experienced by older adults in Lebanon extend beyond the health dimension, as they also face a range of social challenges that can affect their well-being [10]. For instance, older adults often experience a diminished sense of autonomy, and the loss of independence can threaten their dignity, self-esteem, and overall quality of life [22]. Furthermore, loneliness and social isolation are well-documented concerns among older adults, including in Arab countries, where strong family structures have not always been sufficient to protect this population from such experiences [23]. Ageism, defined as stereotypes, prejudice, and discrimination based on age, represents another major challenge. Indeed, high levels of ageism have been documented in Lebanon [24,25]. Older adults are particularly vulnerable to environmental challenges, including natural disasters, climate-related events, and humanitarian crises, which can disrupt essential resources. These events often disproportionately affect vulnerable populations such as older adults, who may face greater difficulties adapting to, responding to, and recovering from such disruptions [26].

These multifaceted challenges underscore the importance of the social aspects of aging and gerontology in Lebanon [27,28]. Understanding the social dimensions of aging is essential for moving beyond solely clinical management, toward holistic care and environments that support independent living for older adults. Holistic aging can be understood as an approach that integrates biomedical care with the psychosocial, community, environmental, and policy dimensions of later life, recognizing that older adults’ health and well-being are shaped as much by their social relationships, living environments, and access to supportive systems as by clinical management alone. This perspective is postulated by conceptualizations of successful and healthy aging that extend beyond the absence of disease to encompass social participation, autonomy, and quality of life within one’s community [29,30]. In Lebanon, however, existing policies and healthcare services for older adults rarely integrate these social dimensions [31]. Current healthcare frameworks in the country remain narrowly focused on a hospital-based model, biomedical care, and nursing homes [18,31,32].

Considering the various challenges experienced by older adults, gerontology and geriatrics research provides a potentially constructive avenue for addressing and mitigating these challenges. Research can be critical to bridge the gap between social or healthcare realities and future practical implications [33]. It is also important since research helps ensure better quality of life and wellbeing for the elderly population, and can help improve health systems and social structures to respond to the needs of this growing demographic [34].

Gerontology and geriatrics are multidimensional fields that include biological, psychological, and social domains, all of which shape quality of life and positive ageing outcomes [3,4]. While the research trends and the important topics for gerontology and geriatrics research globally have been previously mapped [3], to our knowledge, a localized Lebanese mapping of this field remains absent. Such a mapping is important considering the specific context of Lebanon. Examining how late adulthood is studied in research is important for understanding how gerontological research is evolving [35]. Consequently, this research gap calls for an overview of the existing literature to better understand how gerontology and geriatrics research has developed within the Lebanese context, as well as its main areas of strength and limitation. Specifically, given the Lebanese context, there is a critical need to evaluate the extent to which existing scholarship addresses aging from a multifaceted or holistic perspective, including public health, traditional healthcare, community health, psychosocial support, environmental design, and policy frameworks, among other aspects.

Beyond its relevance to Lebanon, this mapping also speaks to a broader and growing global concern. A number of countries, particularly low- and middle-income and crisis-affected regions, are experiencing rapid population aging without the institutional infrastructure, welfare systems, and long-term care capacity that typically support aging in high-income countries. Lebanon’s experience of rapid demographic aging alongside a crisis context and healthcare system fragility offers an instructive case for understanding aging research and framing future research priorities in similar settings. As such, the patterns identified in this review may be relevant not only to Lebanon but also to other countries facing similar circumstances of demographic transition, resource constraints, and instability, helping inform how gerontological research agendas can be more deliberately broadened in these contexts.

Bibliometric analysis offers a suitable methodological approach for this purpose, and can provide an evidence-based foundation for assessing the current state of research and guiding future scholarly development in Lebanon. Thus, this bibliometric review aimed to examine the evolution and trends of gerontology and geriatrics research in Lebanon between 2015 and 2026, map the research landscape by identifying the most productive contributors, collaboration patterns, and thematic areas, and identify notable research gaps to propose priorities for future research and development in the field. Indeed, by mapping the existing literature, this review serves as a resource for aging researchers and policymakers in Lebanon. Overall, the review is guided by the following central questions:

(1) What are the research trends in Lebanese gerontology and geriatrics research?
(2) What are the dominant themes and topics within the literature?
(3) What are the key research gaps in Lebanese gerontology and geriatrics, and what future studies are needed to support holistic aging in Lebanon?

## 2. Materials and Methods

### 2.1. Literature Search

A bibliometric search was conducted using the Scopus database on 10 May 2026 [36]. Scopus was selected because it is one of the largest multidisciplinary databases of peer-reviewed literature and is widely used in bibliometric research due to its extensive coverage and citation indexing capabilities [37,38]. Given the multidisciplinary nature of the fields of gerontology and geriatrics, which encompass health, psychological, social, and policy-related dimensions of aging, among others [39,40,41], Scopus was deemed the most suitable database for capturing the breadth of research conducted in Lebanon.

The search strategy was designed to identify publications related to gerontology, geriatrics, aging, and older adults with at least one author affiliated with a Lebanese institution. The following search query was applied:

(TITLE-ABS-KEY (gerontology) OR TITLE-ABS-KEY (geriatric) OR TITLE-ABS-KEY (geriatrics) OR TITLE-ABS-KEY (older adults) OR TITLE-ABS-KEY (older people) OR TITLE-ABS-KEY (elderly) OR TITLE-ABS-KEY (elder) OR TITLE-ABS-KEY (nursing home) OR TITLE-ABS-KEY (retirement)) AND PUBYEAR > 2014 AND PUBYEAR < 2027 AND (LIMIT-TO (LANGUAGE, “English”) OR LIMIT-TO (LANGUAGE, “French”) OR LIMIT-TO (LANGUAGE, “Arabic”)) AND (LIMIT-TO (AFFILCOUNTRY, “Lebanon”))

This search strategy was designed to identify publications related to gerontology, geriatrics, aging, and older adults. Core terms (gerontology, geriatric, geriatrics, older adults, older people, elderly, elder) were selected as broad, foundational descriptors expected to capture targeted publications in Scopus via either Author Keywords or Index Keywords (retrieved through the TITLE-ABS-KEY field tag). This is similar to broad search strategies employed in previous bibliometric reviews [42,43,44]. Additionally, terms such as nursing home and retirement were included to ensure the retrieval of structural, institutional, or policy-focused publications that might not explicitly center on individual-level aging outcomes or contain the aforementioned keywords.

To delineate geographic scope, the country affiliation filter (AFFILCOUNTRY) was utilized instead of the topic keyword “Lebanon” in titles, abstracts, or keywords, which would have introduced major selection bias. Indeed, many studies conducted in Lebanon may omit the country name from these fields, meaning a keyword-based geographic search would have systematically omitted a substantial portion of local literature. A similar strategy has been used in previous local or regional bibliometric analyses [45,46,47]. This means that the resulting dataset would represent publications retrieved using a defined set of aging-related terms, or a substantial portion, but not the entirety, of Lebanese gerontology and geriatrics research.

Moreover, to assess the accuracy of the search strategy, a brief search sensitivity analysis [48] was conducted comparing the used strategy which relied on a region filter against a geographic topic keyword string (TITLE-ABS-KEY(“leban*” OR “beirut”)). The analysis suggested that the search strategy using the region filter provided optimal recall without omitting relevant literature (see Supplementary Materials).

The search was restricted to publications published between 2015 and 2026 to provide an overview of the most recent decade of research in the field. Only publications written in English, French, or Arabic were considered. Articles in these three languages were included, because they are predominant languages of scientific publication among Arab scholars and researchers [49,50].

### 2.2. Selection and Data Cleaning Process

The retrieved records were exported from Scopus as a comma-separated values (CSV) file. Then, the file was converted into Microsoft Excel to facilitate screening and data cleaning [51]. Duplicate records were first identified and removed.

Subsequently, the remaining records were screened based on their titles, abstracts, and keywords. Since it is a bibliometric review, full texts were not examined [52]. Studies were included if they focused on older adults, aging, gerontology, geriatrics, or geriatric care and were conducted among Lebanese populations or addressed aging-related issues in Lebanon. Specifically, similar to a previous local bibliometric review [51], research in Lebanon was operationally defined as studies conducted with a sample from the country; regional or multinational studies that explicitly reported findings for Lebanon; or policy or theoretical papers directly addressing the Lebanese aging context. The included studies did not need to be done by researchers from Lebanon. Conversely, studies conducted among diaspora populations abroad or studies by local authors conducted exclusively in non-Lebanese settings without addressing aging in Lebanon were excluded.

Since the focus was specifically on gerontology and geriatrics research, studies focusing on other age groups, such as middle adults or young adults, were excluded. Studies based on general population samples were excluded unless it was apparent from the title, abstract, or keywords that analyses or findings pertaining specifically to older adults (typically aged 60 years and above) were reported separately [53]. In this sense, general population studies that did not provide distinct analyses for older adults were excluded. Furthermore, publications involving Lebanese-affiliated authors but conducted among non-Lebanese populations and not addressing aging in Lebanon were excluded.

The screening process was initially conducted by one researcher, after which a second researcher independently reviewed all items and performed a separate eligibility assessment. Discrepancies between the two reviewers were subsequently identified. Disagreements were intended to be resolved through discussion, with any major disagreements to be adjudicated by a third reviewer. Ultimately, all identified discrepancies were successfully resolved through consensus discussion between the two primary researchers, without requiring third-reviewer intervention.

**Figure 1:**
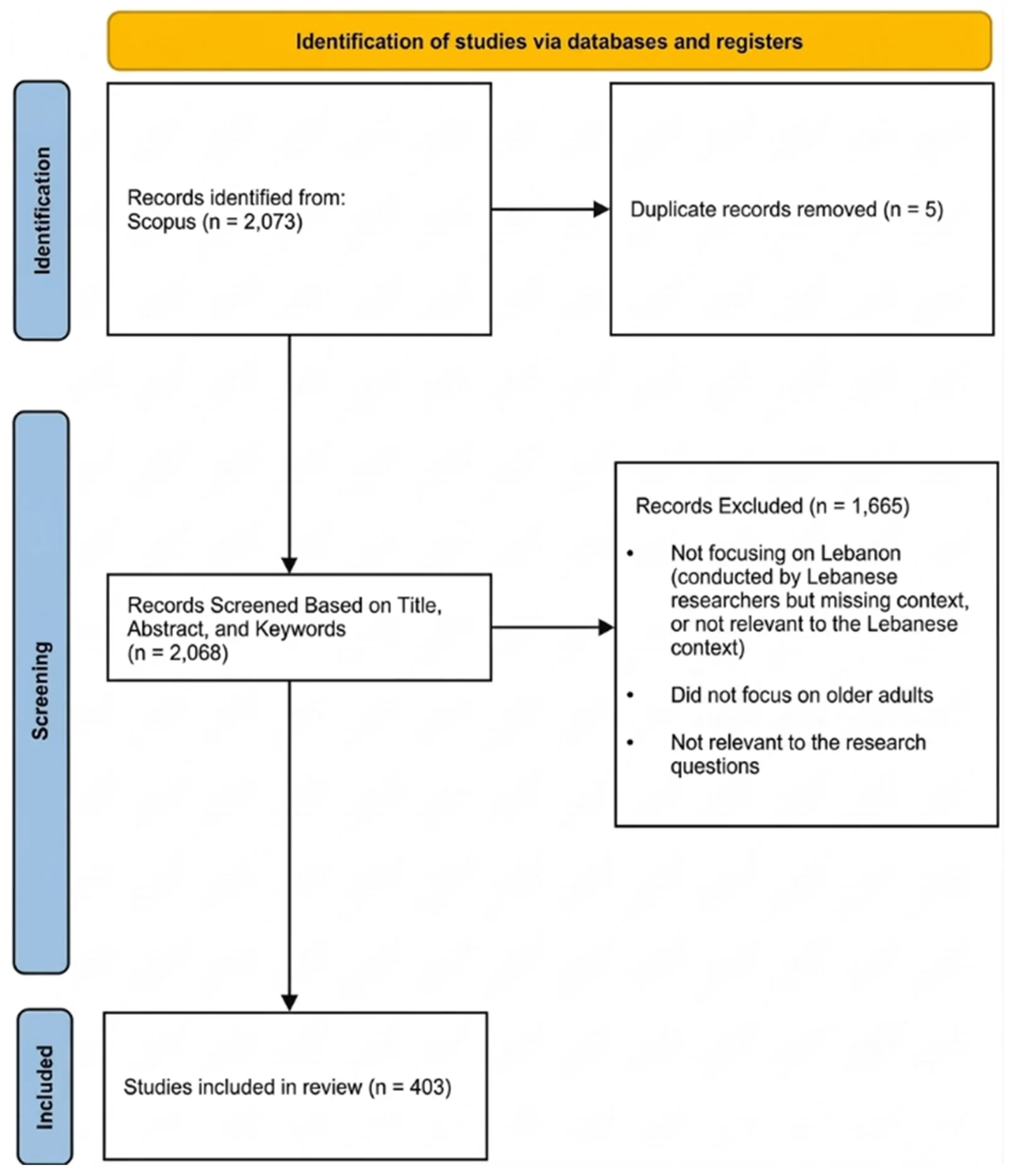
PRISMA Flow Diagram.

The search initially yielded 2073 records. Following duplicate removal and the application of the eligibility criteria, 403 publications were retained for bibliometric analysis. The study selection process is presented in the flow diagram [54] (Figure 1).

### 2.3. Data Analysis

The final dataset was analyzed using Biblioshiny, the web-based interface of the Bibliometrix R package (version 5.4.0.) [55]. Biblioshiny was used to assess annual scientific production, publication growth trends, source productivity, author productivity, institutional productivity, and patterns of national and international collaboration. The software was also used to identify the most frequently occurring keywords and to examine thematic structures and research trends within the field.

To further investigate relationships among research topics, a keyword co-occurrence analysis was conducted using VOSviewer (version 1.6.20) [56]. Network visualization maps were generated to identify major thematic clusters and conceptual linkages within Lebanese gerontology and geriatrics research.

## 3. Results

### 3.1. Research Productivity

The bibliometric analysis covered the period between 1 January 2015 and 10 May 2026. A total of 403 publications from 271 sources were included in the bibliometric analysis. These publications were produced by 1929 authors, of whom only nine authored single-authored publications. The average number of co-authors per document was 6.63, highlighting the collaborative nature of research in the field. International co-authorships accounted for 46.15% of all publications.

**Figure 2:**
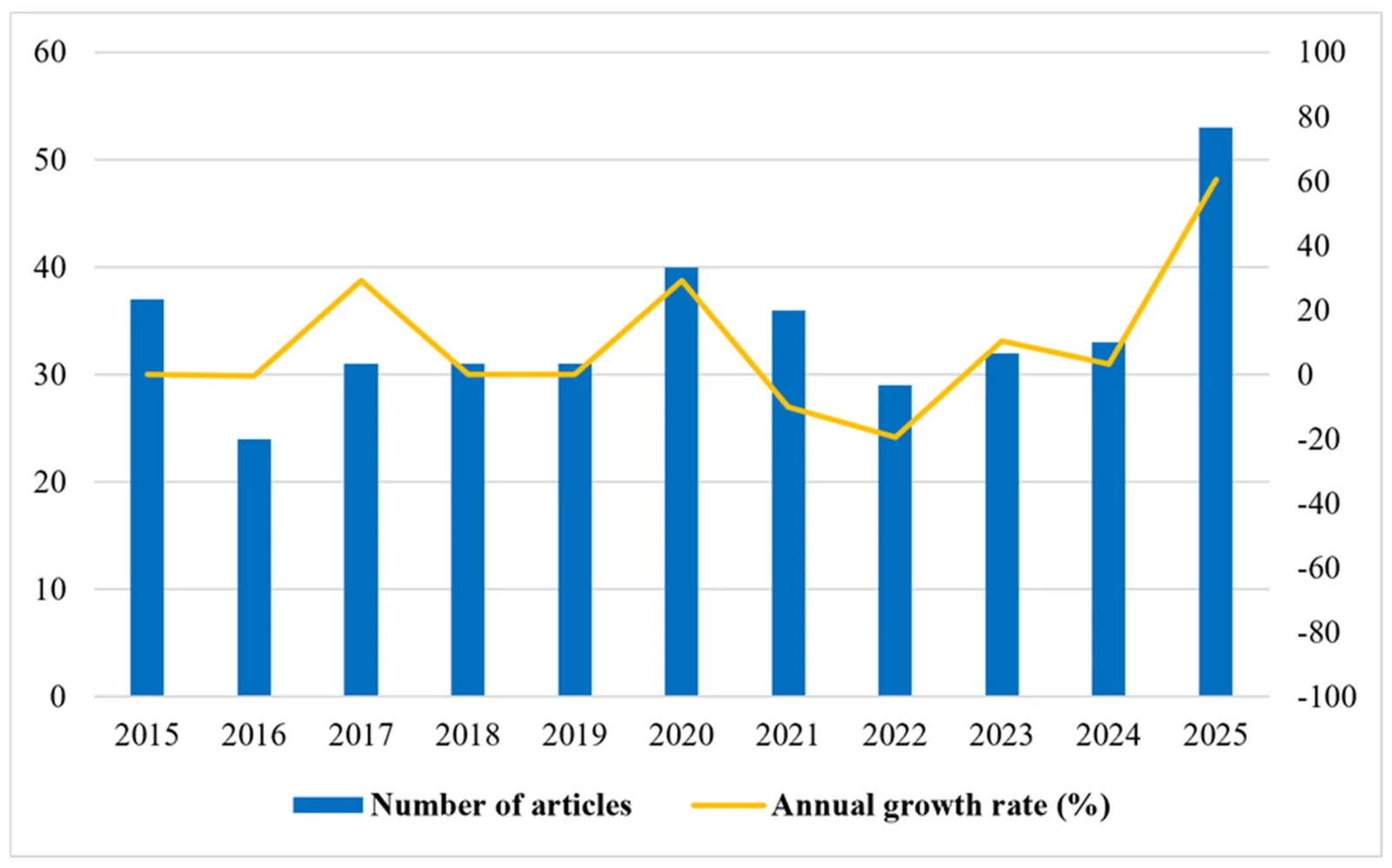
Yearly productivity and growth rate (2015–2025).

Annual scientific production from 2015 to 2025 was steady and generally increasing over the last decade (Figure 2). Publication output remained relatively stable during the earlier years, followed by a gradual increase beginning around 2020. The highest annual productivity was recorded in 2025, with 53 publications, representing the largest number of gerontology and geriatrics related studies produced in a single year in Lebanon during the last decade.

**Figure 3:**
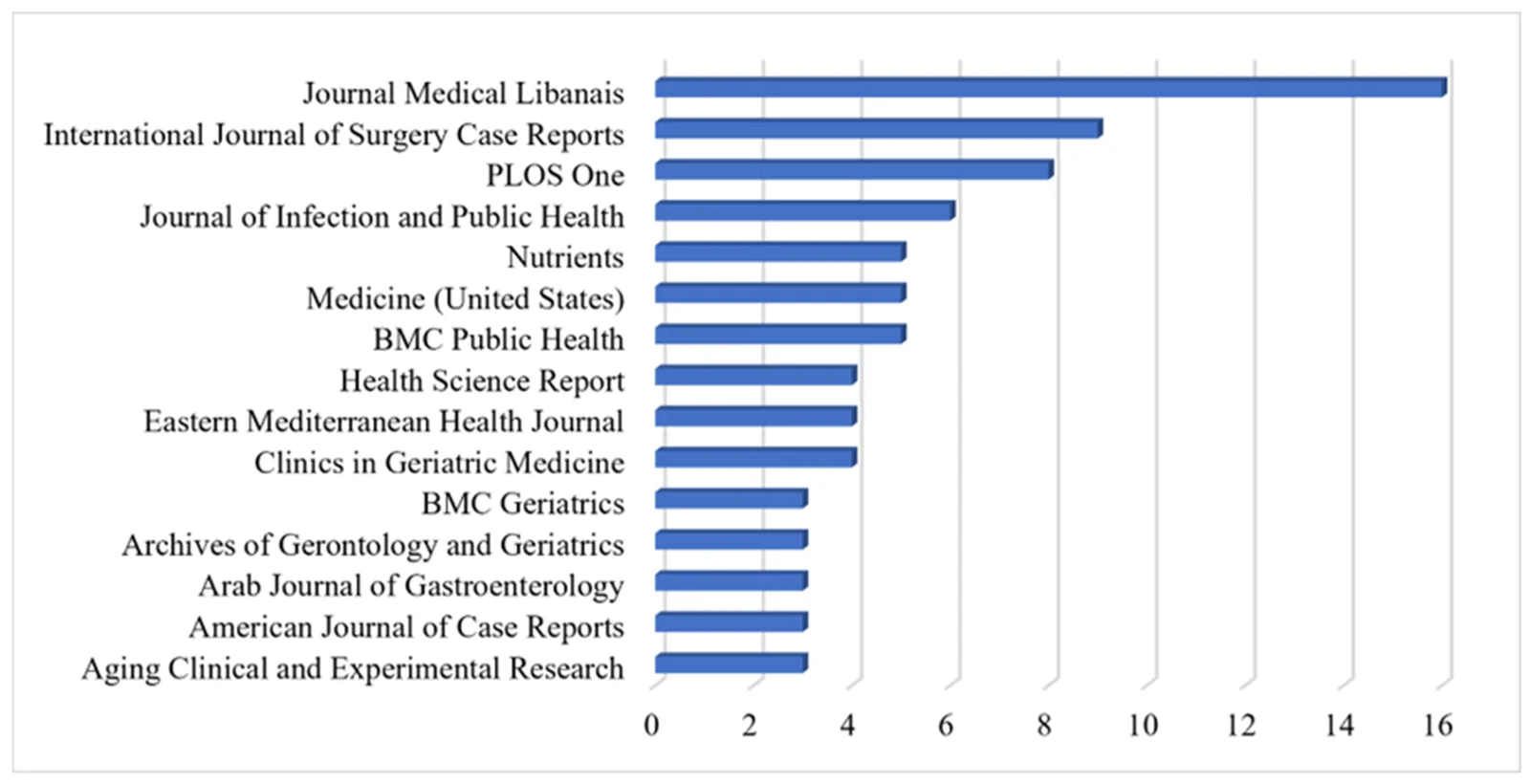
Most published sources.

The most productive source was *Journal Medical Libanais* with 16 publications, followed by *International Journal of Surgery Case Reports* (n = 9), PLOS ONE (n = 8), *Journal of Infection and Public Health* (n = 6), and *BMC Public Health*, *Medicine*, and *Nutrients* (n = 5 each). Most of the leading publication sources were healthcare-oriented journals focusing on clinical medicine, public health, nutrition, and related disciplines. Journals specifically dedicated to broader aging research appeared less frequently among the most productive sources. For example, Archives of Gerontology and Geriatrics ranked lower in terms of publication output (Figure 3).

In terms of single publications, the most cited article was the study by Mokdad et al. [57] on health trends in the Eastern Mediterranean region. This was followed by two studies conducted by Boulos et al. addressing malnutrition among institutionalized and community-dwelling older adults in Lebanon. Several highly cited studies also addressed broader health-system and humanitarian issues, including the Syrian refugee crisis and the health needs of displaced populations in Lebanon. Notably, while certain top-cited studies examine health and nutritional risks within community settings, evaluations of community-based initiatives, interventions, or local support programs remain absent from this highly cited corpus (Table 1).

Among individual researchers, Salameh P was the most productive author with 28 publications. Other leading contributors included Chaaya M (n = 16), Boulos C (n = 14), Hallit S (n = 13), Tamim H (n = 12), and Sibai AM (n = 10). Abdulrahim S, Baddoura R, Chahine B, and El Asmar K each contributed eight publications. Most of these individuals are public health researchers, which suggests that the development of gerontology and geriatrics research in Lebanon is centered on epidemiological and health perspectives.

**Figure 4:**
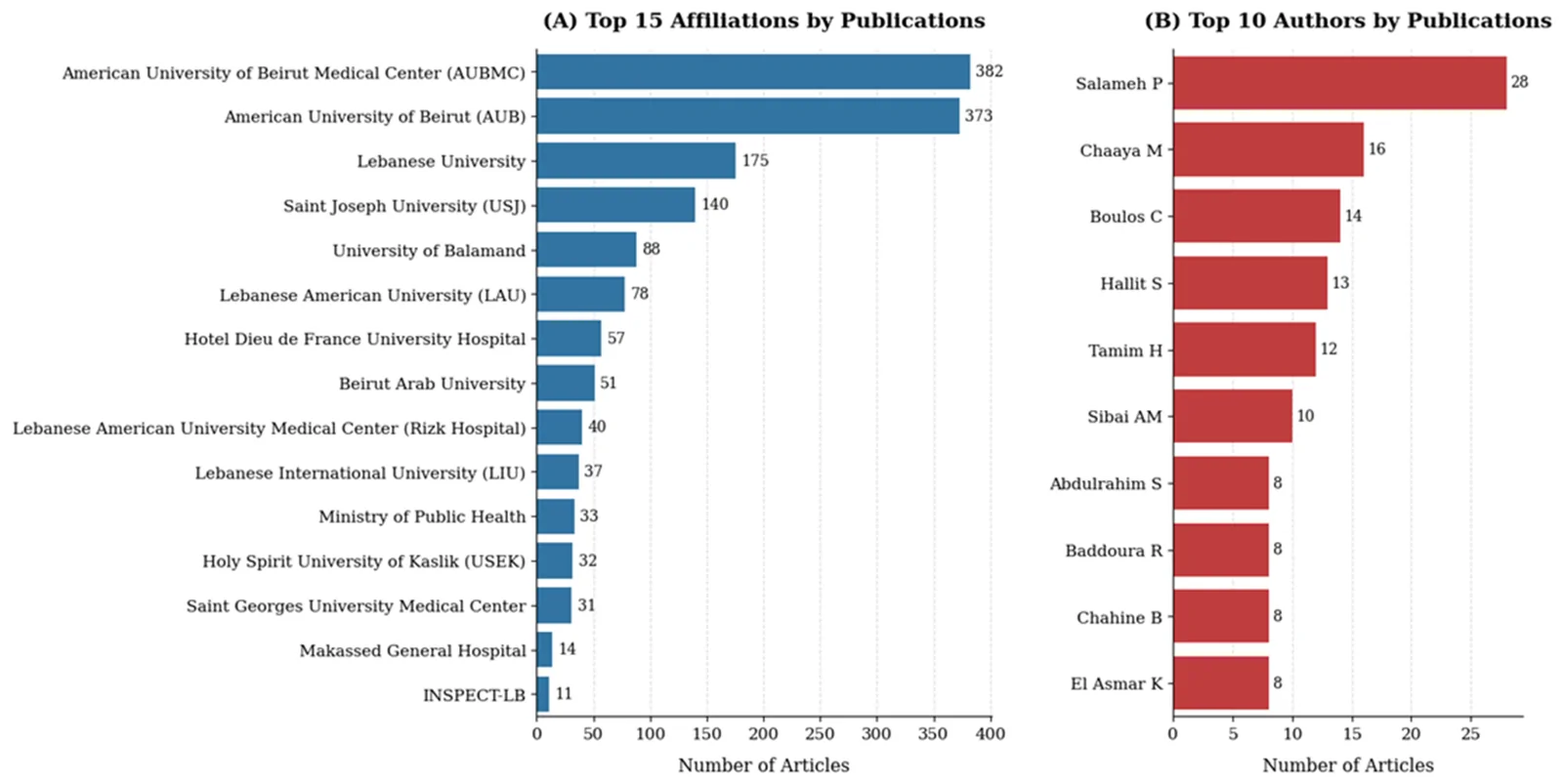
Most productive affiliations and authors.

At the institutional level, the American University of Beirut Medical Center (AUBMC) was the most productive affiliation with 382 article contributions, followed by the American University of Beirut (AUB) with 373 contributions. The Lebanese University ranked third (n = 175), followed by Saint Joseph University (USJ) (n = 140) and the University of Balamand (n = 88). Other notable contributors included the Lebanese American University (LAU) (n = 78), Hôtel-Dieu de France University Hospital (n = 57), Beirut Arab University (n = 51), and the Lebanese American University Medical Center-Rizk Hospital (n = 40). Overall, universities, university medical centers, and healthcare institutions accounted for the majority of research output in the field (Figure 4).

**Figure 5:**
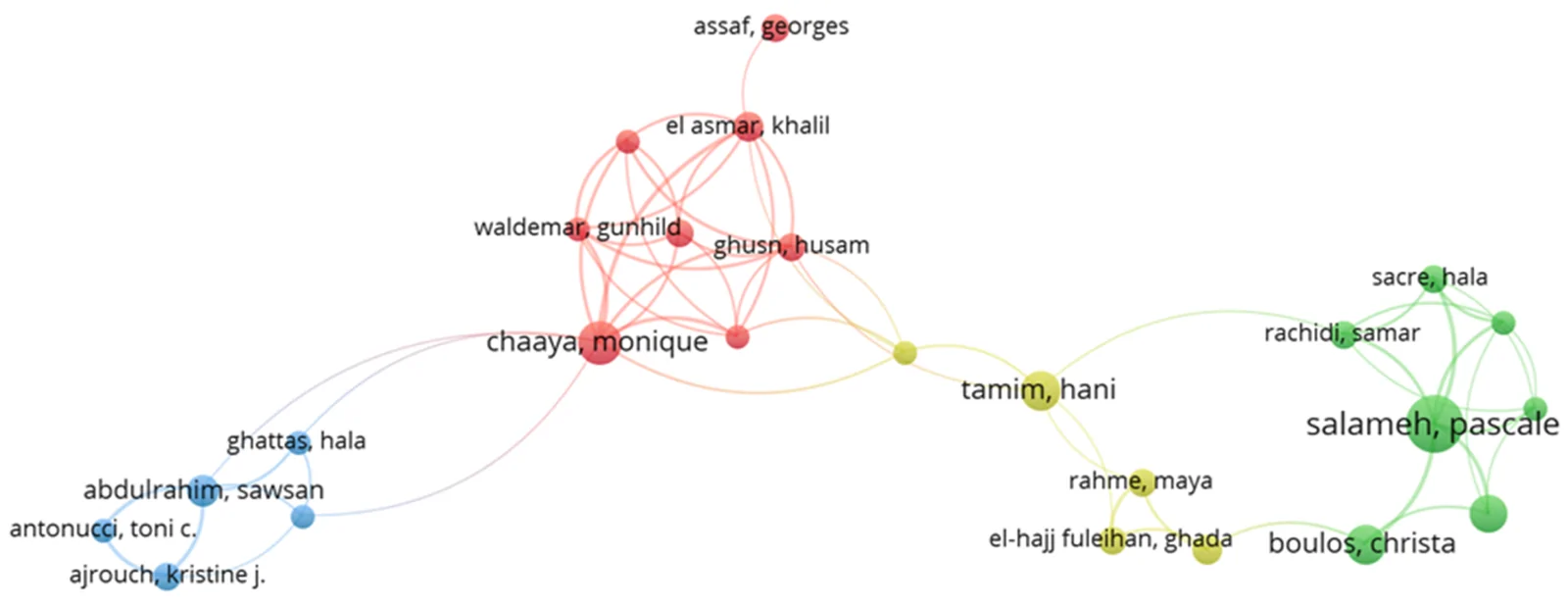
Co-authorship network.

The co-authorship network analysis revealed four main clusters of collaborating researchers that account for a substantial proportion of gerontology and geriatrics research in Lebanon (Figure 5). Each cluster was centered around a group of highly connected authors and reflected recurring patterns of collaboration within the field. While some collaborations occurred between clusters, the network structure suggests that research productivity is largely driven by a limited number of established research groups, with most being public health researchers, epidemiologists, and medical researchers.

International co-authorship represented 46.15% of all publications. The United States was the most frequent international collaborator with Lebanon (n = 67), followed by France (n = 61). The United Kingdom and Jordan each accounted for 15 collaborative publications, followed by Canada (n = 13), the United Arab Emirates (n = 12), and Cyprus, Egypt, and Saudi Arabia (n = 11 each). Denmark completed the top ten collaborating countries with nine publications. These findings indicate extensive international collaboration, particularly with North American, European, and regional Middle Eastern countries (Table 2).

### 3.2. Keyword and Thematic Analysis

The most frequently occurring keywords were “Lebanon” (n = 84), “elderly” (n = 38), “older adults” (n = 30), “aging” (n = 19), “COVID-19” (n = 19), “dementia” (n = 16), “mortality” (n = 15), “depression” (n = 12), “Middle East” (n = 11), “osteoporosis” (n = 11), and “case report” (n = 10). Other prominent keywords included “nursing homes”, osteoporosis”, “palliative care”, “vitamin d”, “epidemiology”, among others. Overall, these dominant keywords are anchored in biomedical markers, clinical pathologies, disease management, and tertiary healthcare settings.

**Figure 6:**
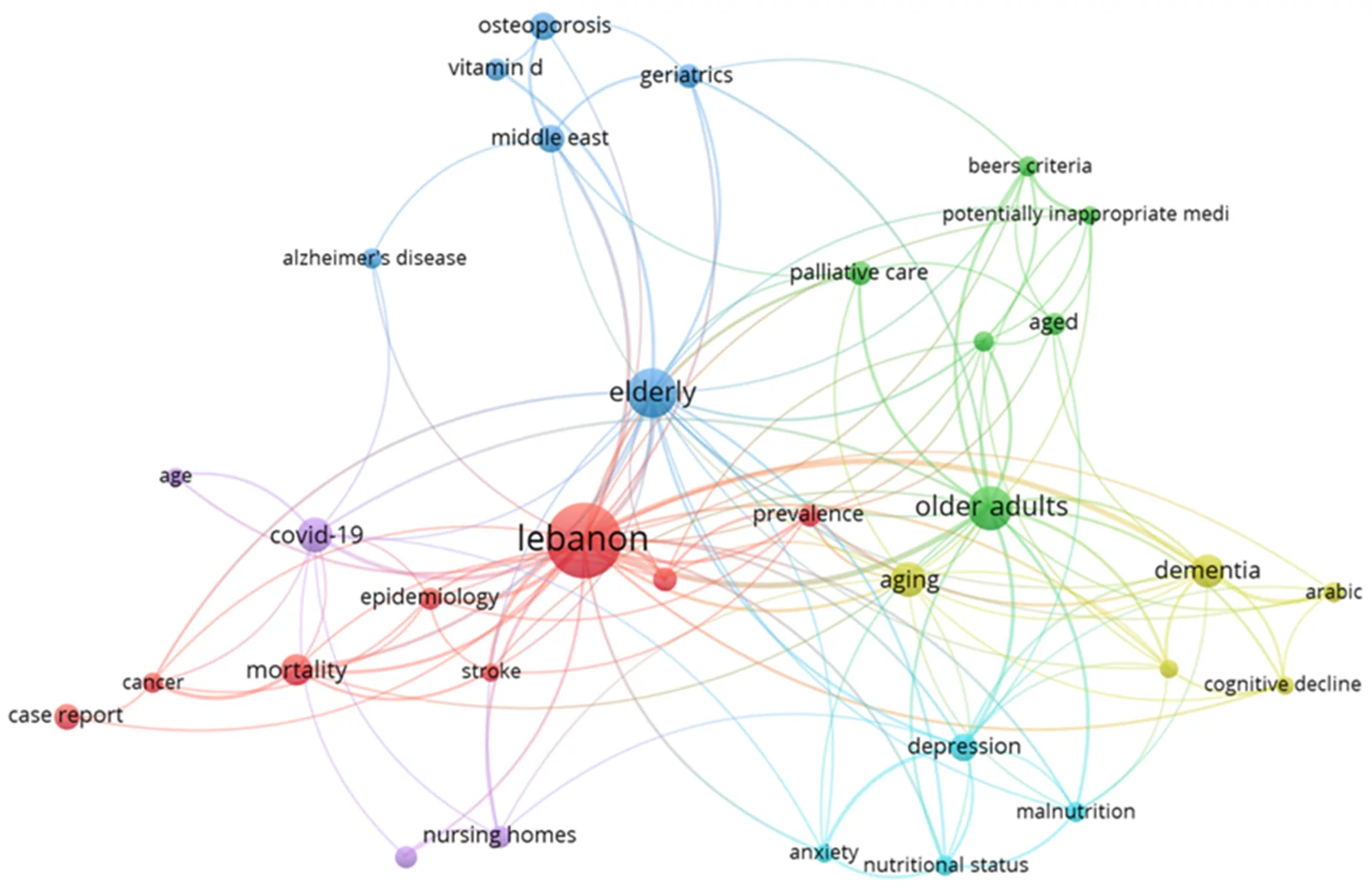
Co-occurrence map of author keywords.

The keyword co-occurrence network identified six thematic clusters (Figure 6). The first cluster centered on epidemiology and public health, while the second focused on nursing homes and social isolation. The third cluster encompassed common health conditions and geriatric syndromes, whereas the fourth cluster was primarily related to mental health and nutrition. The fifth cluster focused on cognitive decline and dementia, and the sixth cluster addressed palliative care and medication-related topics. Across all six identified clusters, the underlying themes mostly revolve around healthcare delivery, disease etiology, and clinical management.

**Figure 7:**
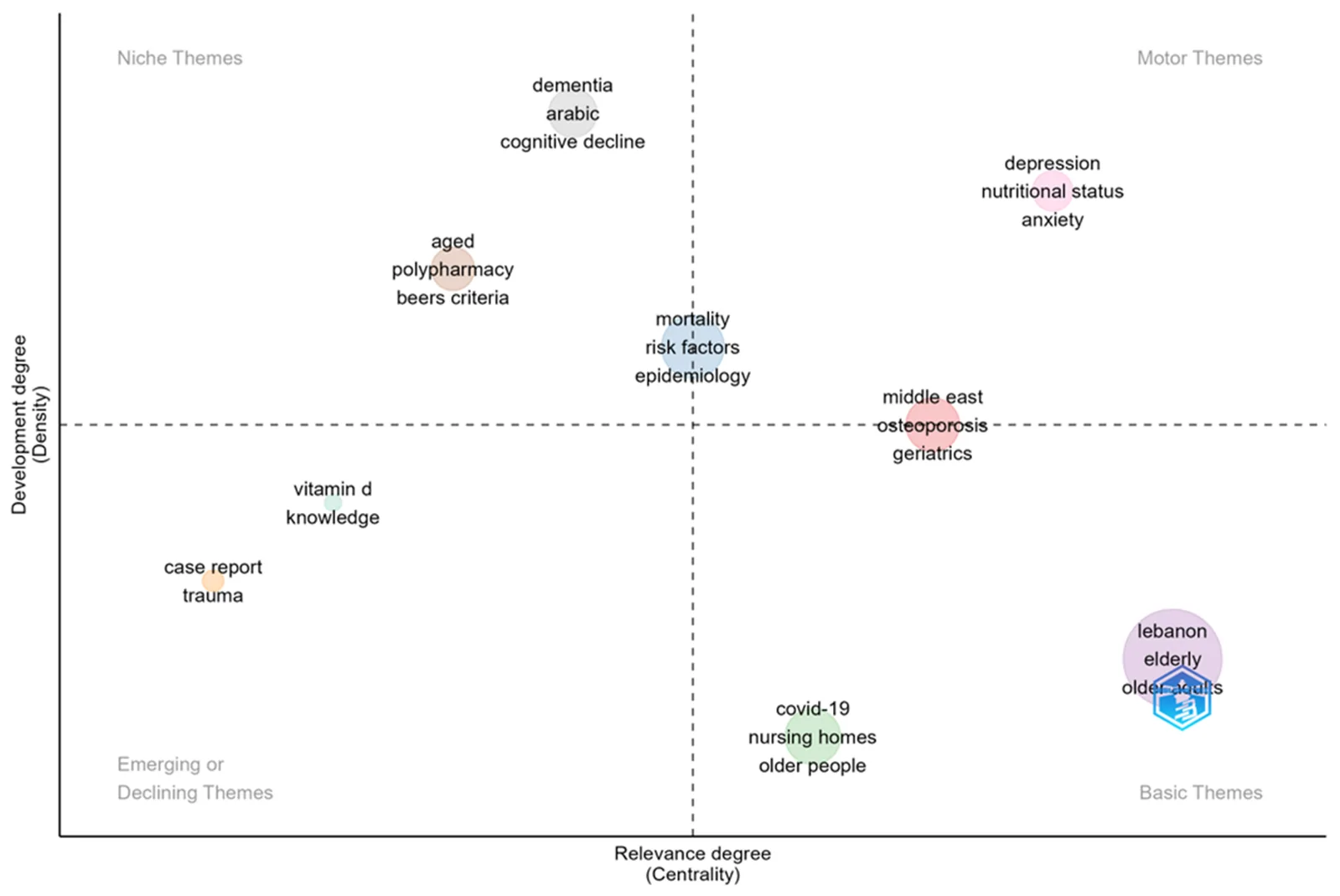
Thematic map of author keywords. Note. Number of keywords = 50. The size of each sphere (circle) is proportional to the occurrence frequency of the keywords within that thematic cluster.

The thematic map classified research themes according to their centrality and density (Figure 7). Motor themes, representing well-developed and highly relevant topics, included terms such as “depression,” “nutritional status,” and “anxiety,” as well as “Middle East,” “osteoporosis,” and “geriatrics.” Basic themes included broad aging-related concepts such as “older adults” and “older people,” alongside “Lebanon,” “COVID-19,” and “nursing homes.”

Niche themes were characterized by high levels of internal development but lower relevance to the overall field and included topics such as “dementia,” “Arabic,” and “cognitive decline,” as well as “aged,” “polypharmacy,” and “Beers criteria.” Emerging or declining themes included topics such as “trauma” and “case report,” as well as “vitamin D” and “knowledge,” reflecting themes that were either newly emerging or becoming less central to the field. The thematic map illustrates the predominance of clinical, epidemiological, and health-related topics, while highlighting several specialized and developing areas of research.

## 4. Discussion: Trends, Gaps, and Future Directions

This bibliometric review examined research in gerontology and geriatrics in Lebanon between 2015 and May 2026. A total of 403 publications were identified and analyzed to characterize research productivity, collaboration patterns, major contributors, and thematic orientations within the field. This review addresses an important gap in the literature, as no previous study has comprehensively mapped gerontology and geriatrics research in Lebanon despite the country’s rapidly aging population. As the proportion of older adults has increased steadily over recent decades, understanding the development, strengths, and shortcomings of the research landscape has become increasingly important for guiding future research, practice, and policy.

### 4.1. Current Research Trends

***a.***  ***Researchers’ and Institutional Productivity***

Over approximately a decade, scientific production remained relatively stable during the earlier years and gradually increased after 2020, reaching its highest level in 2025. This increase coincided with broader international attention to aging and older populations during and after the COVID-19 period [67], during which older adults became a major focus of public health discussions because they experienced heightened social isolation and increased health vulnerabilities [68,69]. In Lebanon specifically, this period also coincided with major socioeconomic and healthcare crises [70,71] that were accompanied by increased attention to vulnerable populations, including older adults [72]. Research during this time of crises in Lebanon has highlighted several barriers affecting healthcare access among older Lebanese adults, including financial constraints, gaps in insurance coverage, and difficulties navigating healthcare services [11]. The growing recognition of these challenges provides a relevant context for understanding the increased research attention in aging and the needs of older adults in the Lebanese context, although the present bibliometric analysis cannot establish the factors underlying the observed increase in publication output.

Despite this growth, it can be argued that publication output remains relatively modest when considered in light of Lebanon’s demographic transition. In fact, previous projections have suggested that adults aged 65 years and older may account for more than one-fifth of the Lebanese population by 2050 [73], which underscores the need to build a stronger evidence base to inform future services, policies, and interventions. This need becomes even more pressing given that more than half of Lebanese older adults lacked health insurance coverage in 2015 [12], further exacerbating barriers to healthcare access and increasing the risk of unmet health needs. Together, these demographic and healthcare realities highlight the importance of expanding gerontology and geriatrics research in Lebanon.

In terms of productivity, research production was concentrated within a limited number of academic and healthcare institutions. AUBMC and AUB represented the most productive affiliations, followed by the Lebanese University and Saint Joseph University. While this concentration is understandable, it may also influence the scope of research priorities. The concentration of publications among institutions with strong health and clinical research programs may partly explain the predominance of biomedical and clinical topics and may provide less coverage of aging experiences outside tertiary healthcare settings. Issues affecting older adults beyond health concerns and institutional healthcare structures tend to receive comparatively less attention. This institutional concentration appears to be reflected in a research paradigm primarily driven by hospital-based research, inpatient care, and pathology management. Community-based studies, such as those addressing home care environments, preventive social interventions, and informal support networks, among others, remain largely secondary within the current academic output in gerontology and geriatrics in Lebanon.

Complementing this, at the author level, productivity was concentrated among a relatively small group of researchers, many of whom are active in public health and health-related disciplines. This pattern suggests that the field of gerontology and geriatrics in Lebanon currently operates primarily as a research area anchored within health sciences, rather than as a fully developed interdisciplinary field. While this reflects a strength in epidemiological and population-health research in Lebanon, gerontology and geriatrics more broadly represent a multidisciplinary domain. In this sense, social, psychological, cultural, and environmental dimensions of aging remain equally important but comparatively less represented.

The collaborative profile of the field was also notable. The average number of co-authors per publication and the considerable proportion of international collaborations indicate that aging research in Lebanon is increasingly integrated into broader scientific networks. The United States and France emerged as the leading collaborating countries, reflecting longstanding academic ties and international partnerships [74]. Such collaborations may strengthen research impact, facilitate resource sharing, enhance scientific visibility, and support the development of local research capacity [75].

***b.***  ***Thematic and Conceptual Trends***

The analysis revealed that the included Lebanese gerontology and geriatrics literature was predominantly concerned with clinical and public health topics. The identified clusters addressed epidemiology and public health, nursing homes and social isolation, common geriatric conditions, mental health and nutrition, cognitive decline and dementia, and palliative and medication-related issues. The predominance of these themes reflects the attention given to major health challenges affecting older adults and is consistent with global priorities in aging research [76,77]. The prominence of topics such as mortality, dementia, osteoporosis, physical performance, and COVID-19 suggests that disease burden, functional decline, and healthcare outcomes were prominent areas of research within the included literature. The thematic map further demonstrated that well-developed and central topics were largely related to geriatrics, epidemiology, and chronic disease management. Topics such as osteoporosis, vitamin D, and physical functioning appeared among the foundational themes identified in the analysis, reflecting substantial attention to physical health and aging outcomes.

These patterns, similar to those of scientific production, also suggest that the field of gerontology and geriatrics in Lebanon mainly operates within health sciences, rather than as an interdisciplinary field. It reflects the significant development of research addressing the health needs of older adults and the growing body of evidence on age-related diseases, functional decline, and healthcare outcomes. However, they also suggest that the identified gerontology and geriatrics research in Lebanon remains largely centered on biomedical concerns, with comparatively less attention devoted to the social, cultural, economic, and policy dimensions of aging, as well as the lived experiences of older adults [39,41,78].

In this sense, the findings suggest that broader or multidisciplinary approaches to aging remain less represented in Lebanese gerontology and geriatrics research. For instance, although themes related to mental health and social isolation appeared in the network analysis, psychosocial, sociological, and environmental aspects of aging received considerably less attention than biomedical topics [39]. This observation is particularly relevant in Lebanon, where aging occurs within a context of economic instability, healthcare strain, repeated societal crises, and age-related stigma [24,70,79]. Indeed, the experience of aging extends beyond disease and healthcare settings to include issues such as social participation, family support, financial security, ageism, living conditions, and access to age-friendly environments. These factors can influence well-being and quality of life in later life [39,80,81,82].

Thus, expanding research toward these dimensions may contribute to a more comprehensive understanding of aging in the Lebanese context. While specialized clinical research remains essential, the future development of the field may benefit from greater integration of medical, psychological, social, and policy perspectives to better reflect the multidimensional nature of aging [39,81,83,84].

### 4.2. Notable Gaps and Implications for Future Research and Policy

***a.***  ***Lack of Research on Community Healthcare for Older Adults***

The following areas represent research opportunities identified from the observed patterns in the present analysis and from considerations raised in the existing literature; they should therefore be interpreted as areas of comparatively limited representation rather than as evidence of an absence of research in Lebanon.

Despite the growing recognition of population aging in the region, community healthcare services designed to enable older adults to age in place were not prominently represented within the literature identified in the present review. This oversight is important given that the vast majority of aging experiences occurs within the context of everyday community life rather than within hospital wards or long-term care facilities. Community-based interventions often focus on maintaining older adults within their own home environments for as long as safely and comfortably possible [85,86]. While international frameworks increasingly emphasize the effectiveness of community-based care models, assistive technologies, and integrated support networks to maintain quality of life in later life [85,86], the literature identified in the present review was primarily focused on medical care and traditional institutional settings. This pattern highlights the comparatively limited representation of research examining how community infrastructure, primary care systems, and informal safety nets can support older adults in their own homes and local environments within the identified literature.

First, relatively little empirical research examining community-based care models tailored to the country was identified in the present review. While family systems remain the primary locus of support [19], structured community healthcare services, such as adult day-care programs, home-visiting nursing initiatives, mobile health clinics, and community care were relatively underrepresented in the identified literature [87]. The identified literature included limited evidence on how these services could be structured or evaluated in relation to premature institutionalization and informal caregiver burden. Understanding the feasibility of integrated community care models is crucial [88], particularly in a context where state-sponsored social protection systems are minimal [12].

Second, the operationalization of ‘aging in place’ was not prominently represented in the literature identified in the present review [89]. The literature provided limited evidence regarding how local neighborhood environments, community safety, housing conditions, and accessibility features shape an older adult’s functional independence and quality of life [90]. Similarly, it provided limited evidence regarding the intersection of physical infrastructure, such as walkability, public transport, and home safety adaptations, with quality of life outcomes among community-dwelling older adults [91].

Third, despite global advances in geron-technology, the role of innovative digital health solutions in promoting independent community living for older adults in Lebanon appears to be underexplored within the identified literature. The bibliometric mapping provides limited evidence in Lebanon regarding research on smart home assistive tools, telehealth platforms, remote vital signs monitoring, and digital safety systems, all of which are increasingly recognized as fundamental pillars of holistic aging in place [92,93,94]. In the Lebanese context, where public health infrastructure is fragmented, digital and assistive technologies could serve as an effective bridge to extend formal and informal support networks directly into the home. However, the identified literature provided limited evidence regarding the feasibility, acceptability, cost-effectiveness, and barriers associated with these interventions [95].

Taken together, these patterns suggest that substantial research opportunities remain in Lebanon to complement the existing focus on clinical care with broader investigations of community-based and holistic approaches to gerontology.

***b.***  ***Lack of Research on Psychosocial Well-being of Older Adults***

Several apparent gaps related to psychosocial well-being can also be identified. For instance, although depression and dementia appear in the literature, aging research in Lebanon seems to conceptualize older adults primarily as a population in need of care and support based on the identified literature. While addressing these vulnerabilities is essential, it is important to recognize that older adulthood is not only a period associated with decline and dependency but also a stage of continued development and personal growth [96]. Also, research examining the effectiveness of psychological and non-pharmacological interventions remains limited, as no clear emphasis on such approaches was apparent in the findings [97,98]. Considering their established benefits in international research, approaches such as reminiscence-based interventions, cognitive-behavioral interventions, psychological support programs, and community-based activities may represent important directions for future research and practice [99,100,101,102,103].

In terms of care settings for older adults, research also appears to be concentrated in nursing homes, as this keyword appears in the co-occurrence map and among the most frequent author keywords. In comparison, less attention is given to alternative models of care, as these do not clearly emerge in the results. Given the central role of family caregiving in Lebanon, where older adults are often supported within multigenerational households [24,104], further investigation of home-based care, community support systems, day-care services, and integrated care models may help inform more contextually appropriate service planning and reduce reliance on institutional care [101,105].

A notable gap is the limited representation of social and sociological dimensions of aging [39]. Indeed, topics such as ageism, elder maltreatment, loneliness, attitudes toward older adults, and social participation appear to be relatively less represented in the identified literature despite their well-established importance in international research [106,107,108,109]. They may be especially relevant in Lebanon given ongoing social change, economic strain, and evolving family structures that may weaken traditional support systems for older adults [79,110].

Furthermore, aging in contexts of conflict, displacement, economic instability, and environmental change appears to have received limited attention in the identified literature, despite these being the specific social challenges the country has been facing for years [111,112]. The country’s history of war, political instability, and recent economic collapse places older adults in particularly vulnerable positions, as these stressors can exacerbate health risks, limit access to services [113].

Environmental gerontology, which examines how climate change, housing conditions, infrastructure, and the built environment influence aging, remains minimally represented in the identified literature despite its growing international relevance for age-friendly planning [114,115]. This area is particularly relevant in Lebanon, where environmental challenges, including pollution, inadequate infrastructure, and climate-related risks, increasingly affect living conditions and can have important implications for older adults’ health and well-being [116].

Lastly, the social policy perspective in gerontology and geriatrics research remains limited in the identified literature. This area of research is highly needed [117], as the identified studies in Lebanon seem to rarely examine how social policies shape aging experiences or how systems of protection, pensions, healthcare coverage, and social assistance respond to the needs of older adults. Strengthening this area of research could help bridge the gap between evidence and policy by informing the development of more inclusive and sustainable aging policies [118]. This is particularly important in the Lebanese context, where large policy gaps exist, especially ones related to older adults [119].

***c.***  ***Biomedical Research Gaps***

While the most notable gaps where on the psychological, social, and community level, some health-related gaps can be noted. Several geriatric syndromes and functional difficulties appeared to be underrepresented in the identified literature. For instance, topics related to mobility limitations, falls, and rehabilitation interventions received limited attention despite their importance for maintaining independence and quality of life in older age [120,121]. Greater emphasis on prevention, rehabilitation, and the preservation of functional capacity may support healthier aging trajectories in Lebanon [122].

Similarly, sensory health received relatively little attention in the identified literature. Vision and hearing impairments are common among older adults and have been associated with reduced autonomy and lower quality of life [123,124]. Research examining the prevalence, consequences, and management of sensory impairments among Lebanese older adults may therefore be valuable.

Sleep-related issues were also largely absent. Conditions such as insomnia can affect physical functioning, mental health, and cognitive outcomes and may represent an important area for future investigation [125,126,127]. Likewise, urinary incontinence and other related geriatric syndromes received minimal attention in the identified literature, despite their potential effects on quality of life [128,129].

**Figure 8:**
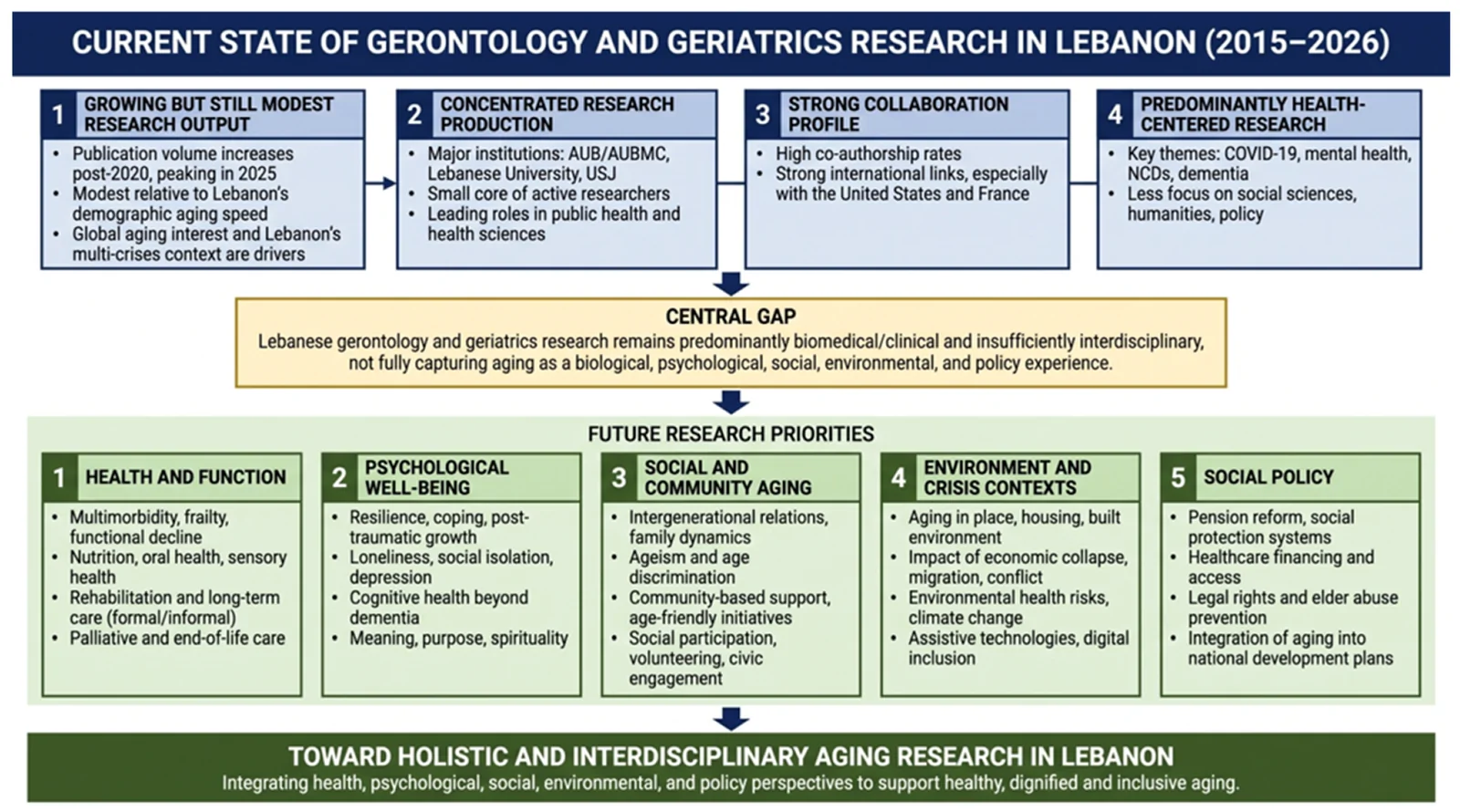
Current State and Future Priorities for Gerontology and Geriatrics Research in Lebanon.

All bibliometric findings, as well as the multidimensional research gaps and suggested future direction are summarized in Figure 8.

### 4.3. Limitations of the Review

Several limitations should be acknowledged. First, this review relied exclusively on the Scopus database. Although this database is one of the largest academic databases, offers extensive multidisciplinary coverage, and is widely used in bibliometric analyses, relevant publications indexed elsewhere or published in regional and non-indexed outlets may not have been captured [130]. Moreover, this means that grey literature, such as governmental policy reports, non-governmental organization evaluations, and other papers, was not explored. Gerontological and geriatric interventions may have been documented outside traditional peer-reviewed journals, meaning that incorporating grey literature could provide additional insights, particularly ones related to practices and policies.

Second, the scope of the review is inherently constrained by the selected search strategy and keywords. Despite efforts to ensure a comprehensive search, it is possible that some relevant studies were not captured due to variations in terminology or indexing practices. As noted in previous research, search strategies can influence the scope and outcomes of reviews, potentially leading to the omission of pertinent literature [131]. Also, while our search strategy was designed to be comprehensive, syntax and keyword constraints mean that the retrieved dataset captures a substantial proportion of Lebanese gerontology and geriatrics research rather than the entire literature. Despite utilizing broad terms and database-assigned index keywords, specialized studies targeting narrow clinical sub-conditions (e.g., rare neurodegenerative disorders or specific metabolic syndromes) that may have lacked demographic or aging keywords may not have been retrieved. Similarly, since terms such as aging in place, age-friendly environments, home care, and community-dwelling, among others, were not included as explicit search terms, studies addressing these specific concepts may not have been retrieved even where relevant Lebanese literature exists. Thus, the thematic patterns discussed in this review, including areas described as comparatively underrepresented, should be interpreted in light of this search constraint, rather than as confirmed evidence that these areas are genuinely absent in the broader Lebanese literature.

Furthermore, relying on database country affiliation filters (AFFILCOUNTRY) excluded studies focused on older populations in Lebanon conducted entirely by international entities without local co-authorship. Nonetheless, as demonstrated by our brief sensitivity analysis, the impact of these constraints was minimal, and the used search strategy provided the most methodologically feasible approach for mapping the field in Lebanon, though this limitation should still be kept in mind when interpreting the findings.

Third, given that this was a bibliometric review aiming to investigate trends in a research field, the literature screening was done based on titles, abstracts, and keywords rather than full-text assessment [51]. Consequently, some publications may have been misclassified or excluded despite relevance to aging research.

Finally, bibliometric findings provide an overview of a research domain but do not directly evaluate methodological quality or practical impact. Therefore, the findings should be interpreted as an overview of research trends rather than an assessment of evidence quality.

## 5. Conclusions

This bibliometric review provides a comprehensive overview of research on gerontology and geriatrics in Lebanon between 2015 and 2026. The field is characterized by strong institutional concentration, a limited number of highly productive authors, and considerable international collaboration.

However, the identified literature was predominantly centered on disease pathologies, epidemiology, and tertiary hospital or nursing home care. In contrast, community-based health services, age-friendly environments, assistive technologies, informal caregiving systems, and social policy frameworks were comparatively less represented in the identified literature. These patterns highlight several areas that warrant greater attention in future Lebanese gerontology and geriatrics research, particularly community-based approaches to supporting older adults in their own communities and the role of assistive and digital technologies in supporting independent living.

Thus, future research may prioritize the development and evaluation of community-based care models, research on aging in place and age-friendly environments, and investigation of assistive and digital health technologies for older adults. These priorities are derived from the comparatively limited representation of these areas in the identified literature and may help broaden the scope of Lebanese gerontology and geriatrics research beyond its current emphasis on clinical and institutional care.

Addressing these areas may contribute to expanding gerontology and geriatrics in Lebanon toward a more multidisciplinary field. Integrating clinical research with psychosocial, community, environmental, and technological perspectives may provide a broader evidence base for researchers, practitioners, and policymakers to develop context-appropriate strategies that support the health, safety, independence, and well-being of older adults.

## Figures and Tables

**Table 1:** Most cited publications.

Authors and Year	Article Title	Journal	Citations
Mokdad et al. (2016) [57]	Health in times of uncertainty in the eastern Mediterranean region, 1990–2013: a systematic analysis for the Global Burden of Disease Study 2013	The Lancet Global Health	180
Boulos et al. (2016) [58]	Prevalence of malnutrition and its correlates in older adults living in long stay institutions situated in Beirut, Lebanon	Clinical Nutrition	153
Boulos et al. (2017) [59]	Factors associated with poor nutritional status among community dwelling Lebanese elderly subjects living in rural areas: results of the AMEL study	Geriatrics & Gerontology International	141
Strong et al. (2015) [60]	Health status and health needs of older refugees from Syria in Lebanon	Conflict and Health	133
Akik et al. (2019) [61]	Host country responses to non-communicable diseases amongst Syrian refugees: a review	Conflict and Health	84
Kabbara et al. (2018) [62]	Reduced integration and improved segregation of functional brain networks in Alzheimer’s disease	Journal of Neural Engineering	75
El Hajj et al. (2019) [63]	Vitamin D supplementation and muscle strength in pre-sarcopenic elderly Lebanese people: a randomized controlled trial	Archives of Osteoporosis	73
Farah et al. (2015) [64]	Antibiotic dispensation by Lebanese pharmacists: a comparison of higher and lower socio-economic levels	Journal of Infection and Public Health	70
Zgheib et al. (2018) [65]	Short Telomere Length is Associated with Aging, Central Obesity, Poor Sleep and Hypertension in Lebanese Individuals	Aging and Disease	68
Darwish et al. (2018) [66]	Cognitive function, vitamin D status, and inflammatory markers in Lebanese older adults	Frontiers in Aging Neuroscience	57

**Table 2:** Most frequent international collaborations.

From	To	Frequency
Lebanon	United States	67
Lebanon	France	61
Lebanon	Jordan	15
Lebanon	United Kingdom	15
Lebanon	Canada	13
Lebanon	United Arab Emirates	12
Lebanon	Cyprus	11
Lebanon	Egypt	11
Lebanon	Saudi Arabia	11
Lebanon	Denmark	9

## Data Availability

The data and its analysis are openly available in Open Science Framework (OSF) at https://doi.org/10.17605/OSF.IO/DCBE9.

## Supplementary Materials

The following supporting information can be downloaded at: https://www.mdpi.com/article/10.3390/healthcare14172857/s1, Supplementary File: Brief Sensitivity Analysis. (Supplementary Table S1: Brief Sentiment Analysis. Supplementary Table S2: Screening Results for Non-Overlapping Records (Search C) [132,133,134,135,136,137,138,139,140,141,142,143]).

## References

[1] Guseh J.S., Shehan C.L. (2015). Aging of the World’s Population. Encyclopedia of Family Studies.

[2] Shrivastava P., Syed Sabir Ali S.S.A. (2025). Analyzing the impact of aging populations on healthcare system sustainability. J. Neonatal Surg..

[3] Neto L.S.S., Rosa T.D.S., Freire M.D., De Luca Correa H., Pedreira R.C., Dias F.C.F., De Oliveira D.V., Osório N.B. (2023). Geriatric and Gerontology Research: A Scientometric Investigation of Open Access Journal Articles Indexed in the Scopus Database. Ann. Geriatr. Med. Res..

[4] Tohit N.F.M., Haque M. (2024). The New Frontier of Ageing: Innovations and Insights in Gerontology. Adv. Hum. Biol..

[5] Abyad A. (1995). Geriatrics in Lebanon: The Beginning. Int. J. Aging Hum. Dev..

[6] Abyad A. (2001). Health Care for Older Persons: A Country Profile—Lebanon. J. Am. Geriatr. Soc..

[7] Central Administration of Statistics (2012). Population and Housing Characteristics in Lebanon.

[8] Khoury R., Karam G. (2020). Impact of COVID-19 on mental healthcare of older adults: Insights from Lebanon (Middle East). Int. Psychogeriatr..

[9] Sibai A.M., Rizk A., Kronfol N.M. (2015). Aging in Lebanon: Perils and Prospects. Leban. Med. J..

[10] Abdulrahim S., Ajrouch K.J., Antonucci T.C. (2015). Aging in Lebanon: Challenges and Opportunities. Gerontologist.

[11] Dableh S., Frazer K., Azar M., Hamadeh R., Kroll T. (2025). Maximizing older people’s access to primary health care centers in Lebanon: A co-design approach. Health Promot. Int..

[12] El Osta N., Hennequin M., El Osta L., Bou Abboud Naaman N., Geahchan N., Tubert-Jeannin S. (2015). État des lieux sanitaire et bucco-dentaire de la population gériatrique libanaise. East. Mediterr. Health J..

[13] Sharpe L., Sweeny K., Robbins M.L., Cohen L.M. (2020). Older Adults and Perspectives for Researchers and Clinicians Working in Health Psychology and Behavioral Medicine. The Wiley Encyclopedia of Health Psychology.

[14] Hoogendijk E., Dent E. (2023). Recent developments in frailty identification, treatment, and prevention in older adults: A review. Innov. Aging.

[15] Hu X., Yu S.-J., Gao Y.-C., Zhao Y., He Y.-S., Liu Y.-C., Pan H.-F., Wang P. (2025). Health inequality in the disease burden of non-communicable diseases among the elderly from 1990 to 2021, and projections to 2050: A systematic analysis of global burden of disease study. BMC Geriatr..

[16] (2022). GBD 2019 Ageing Collaborators Global, regional, and national burden of diseases and injuries for adults 70 years and older: Systematic analysis for the Global Burden of Disease 2019 Study. BMJ.

[17] Jalali A., Ziapour A., Karimi Z., Rezaei M., Emami B., Kalhori R.P., Khosravi F., Sameni J.S., Kazeminia M. (2024). Global prevalence of depression, anxiety, and stress in the elderly population: A systematic review and meta-analysis. BMC Geriatr..

[18] Noubani A., Diaconu K., Loffreda G., Saleh S. (2021). Readiness to deliver person-focused care in a fragile situation: The case of Mental Health Services in Lebanon. Int. J. Ment. Health Syst..

[19] Barakat Z., Sacre H., El Khatib S., Abou Abbas L., Barakat M., Salameh P., Rachidi S. (2026). Coping strategies among family caregivers of community-dwelling older adults in Lebanon amid the economic crisis. PLoS ONE.

[20] Greenfield E.A., Black K., Buffel T., Yeh J. (2019). Community Gerontology: A Framework for Research, Policy, and Practice on Communities and Aging. Gerontologist.

[21] Ganesh A., Rajan S.I. (2025). Community-Based Healthcare of the Elderly. Handbook of Aging, Health and Public Policy.

[22] Raja M., Uhrenfeldt L., Galvin K.T., Kymre I.G. (2022). Older adults’ sense of dignity in digitally led healthcare. Nurs. Ethics.

[23] Almaimani H.A., Moafa W.A., Aqili T.A., Homadi S.Y. (2024). A Cross-Sectional Study on Social Isolation and Loneliness Related to COVID-19 Among Middle and Late-Stage Elderly in Jazan, Saudi Arabia. Cureus.

[24] Younes R.S., Abboud Mzawak M. (2026). Ageism and Attitudes Toward Older Adults in Arab Culture: A Systematic Integrative Review. Soc. Sci..

[25] Younes R.S., Rajha F. (2025). Attitudes toward older adults among Arab healthcare professionals and students: A systematic review and recommendations for geriatric care. NPG Neurol. Psychiatr. Gériatr..

[26] Mariscal Ureta K.E., Martínez Nieves G.E. (2026). Vulnerabilidad, Crisis Ambiental y Derechos Humanos. Ibero Cienc. Rev. Cient. Acad..

[27] Bhugra D., Liebrenz M., Smith A. (2026). Mental health in the time of polycrisis: Geopolitical determinants and modern psychiatry. Front. Psychiatry.

[28] El-Khoury J., Haidar R., Charara R. (2020). Community mental healthcare in Lebanon. Consort. Psychiatr..

[29] Keating N. (2022). A research framework for the United Nations Decade of Healthy Ageing (2021–2030). Eur. J. Ageing.

[30] Horgan S., Prorok J., Ellis K., Mullaly L., Cassidy K.-L., Seitz D., Checkland C. (2024). Optimizing Older Adult Mental Health in Support of Healthy Ageing: A Pluralistic Framework to Inform Transformative Change across Community and Healthcare Domains. Int. J. Environ. Res. Public Health.

[31] Porcel-Gálvez A.M., Allande-Cussó R., Fernández-García E., Essawi S., Salama M., Saad S.S., Fernández-Castillo R.-J., Serrano M.L. (2024). Priorities and challenges in social and healthcare policies for older people living in the Mediterranean basin: A Delphi panel study. BMC Geriatr..

[32] Aoun N., Tajvar M. (2024). Healthcare delivery in Lebanon: A critical scoping review of strengths, weaknesses, opportunities, and threats. BMC Health Serv. Res..

[33] Lim W.M., Bowman C. (2023). How to establish practical contributions and convey practical implications? Guidelines on locating practice gaps and making recommendations for practice. Act. Adapt. Aging.

[34] Singh S., Yadav R., Doskaliuk B. (2023). Aging and geriatric care: A global imperative towards universal health coverage. Anti-Aging East. Eur..

[35] Zheng Z., M. H.-S.B.A., Zaki H.O., Tan Q.L. (2025). Aging and Its Societal Impact: A Bibliometric Analysis. SAGE Open.

[36] Passas I. (2024). Bibliometric Analysis: The Main Steps. Encyclopedia.

[37] Baas J., Schotten M., Plume A., Côté G., Karimi R. (2020). Scopus as a curated, high-quality bibliometric data source for academic research in quantitative science studies. Quant. Sci. Stud..

[38] Burnham J.F. (2006). Scopus database: A review. Biomed. Digit. Libr..

[39] Higgs P., Gilleard C. (2023). The sociology of aging and social gerontology: Critical tensions and necessary distinctions. Innov. Aging.

[40] Musselwhite C. (2025). Gerontology and geriatrics: Care and context. Towards Integration. Cogent Gerontol..

[41] Younes R.S., Alayan M., Rahme E. (2026). Reconceptualizing successful aging through a psychodynamic and psychoanalytic perspective: Towards an integrative approach in gerontology. NPG Neurol. Psychiatr. Gériatr..

[42] Pan X., Liang B., Cao T. (2024). A bibliometric analysis of speech and language impairments in Parkinson’s disease based on Web of Science. Front. Psychol..

[43] Sater R.A., Rayess Y.E., Julien S.G. (2026). Oxidative stress as a potential mechanistic bridge between electronic cigarette components and chronic disease: A combined narrative and bibliometric analysis. Antioxidants.

[44] Nehmé L., Tekle M.E., Barakat N., Khoury A.E., Azzi-Achkouty S., Rayess Y.E. (2024). Alternative Processes for apple Juice stabilization and clarification: A Bibliometric and Comprehensive review. Processes.

[45] Abdelwahab S.I., Taha M.M.E., Farasani A., Abdullah S.M., Moshi J.M., Khamjan N.A., Assiri A., Alshahrani S., Sahli K.A., Farasani M.M. (2025). South Asia’s neuroscience research: Publication volume, quality metrics, and international comparisons (2020–2025). NPG Neurol. Psychiatr. Geriatr..

[46] Dardas L.A., Malkawi A.M.A., Sweis S., Sweis N., Al-Khayat A., Sawair F.A. (2023). Mapping Two Decades of Research Productivity in the Middle Eastern and Arab Countries: A Comprehensive Bibliometric Analysis. Publications.

[47] Bissar-Tadmouri N., Tadmouri G.O. (2009). Bibliometric analyses of biomedical research outputs in Lebanon and the United Arab Emirates 1988–2007. Saudi Med. J..

[48] Lagisz M., Yang Y., Young S., Nakagawa S. (2025). A practical guide to evaluating sensitivity of literature search strings for systematic reviews using relative recall. Res. Synth. Methods.

[49] Ashoor M.S., Chaudhry A.S. (1993). Publication Patterns of Scientists Working in Saudi Arabia. Int. Inf. Libr. Rev..

[50] Elgamri A., Mohammed Z., El-Rhazi K., Shahrouri M., Ahram M., Al-Abbas A.-M., Silverman H. (2024). Challenges facing Arab researchers in conducting and publishing scientific research: A qualitative interview study. Res. Ethics.

[51] Hajj A., Sacre H., Ounsi M., Nasser M., Mohsen Meame S., Hazime H., Al-Hajje A., Rahme D., Lahoud N., Safwan J. (2025). Antimicrobial research productivity in Lebanon: A PubMed-Based bibliometric analysis. New Microbes New Infect..

[52] Öztürk O., Kocaman R., Kanbach D.K. (2024). How to design bibliometric research: An overview and a framework proposal. Rev. Manag. Sci..

[53] United Nations (2017). World Population Ageing.

[54] Page M.J., McKenzie J.E., Bossuyt P.M., Boutron I., Hoffmann T.C., Mulrow C.D., Shamseer L., Tetzlaff J.M., Akl E.A., Brennan S.E. (2021). The PRISMA 2020 statement: An updated guideline for reporting systematic reviews. BMJ.

[55] Aria M., Cuccurullo C. (2017). bibliometrix: An R-tool for comprehensive science mapping analysis. J. Informetr..

[56] Van Eck N.J., Waltman L. (2010). Software survey: VOSviewer, a computer program for bibliometric mapping. Scientometrics.

[57] Mokdad A.H., Forouzanfar M.H., Daoud F., Bcheraoui C.E., Moradi-Lakeh M., Khalil I., Afshin A., Tuffaha M., Charara R., Barber R.M. (2016). Health in times of uncertainty in the eastern Mediterranean region, 1990–2013: A systematic analysis for the Global Burden of Disease Study 2013. Lancet Glob. Health.

[58] El Zoghbi M., Boulos C., Awada S., Rachidi S., Al-Hajje A., Bawab W., Saleh N., Salameh P. (2014). Prevalence of malnutrition and its correlates in older adults living in long stay institutions situated in Beirut, Lebanon. J. Res. Health Sci..

[59] Boulos C., Salameh P., Barberger-Gateau P. (2014). Factors associated with poor nutritional status among community dwelling Lebanese elderly subjects living in rural areas: Results of the AMEL study. J. Nutr. Health Aging.

[60] Strong J., Varady C., Chahda N., Doocy S., Burnham G. (2015). Health status and health needs of older refugees from Syria in Lebanon. Confl. Health.

[61] Akik C., Ghattas H., Mesmar S., Rabkin M., El-Sadr W.M., Fouad F.M. (2019). Host country responses to non-communicable diseases amongst Syrian refugees: A review. Confl. Health.

[62] Kabbara A., Eid H., Falou W.E., Khalil M., Wendling F., Hassan M. (2018). Reduced integration and improved segregation of functional brain networks in Alzheimer’s disease. J. Neural Eng..

[63] Hajj C.E., Fares S., Chardigny J.M., Boirie Y., Walrand S. (2018). Vitamin D supplementation and muscle strength in pre-sarcopenic elderly Lebanese people: A randomized controlled trial. Arch. Osteoporos..

[64] Farah R., Lahoud N., Salameh P., Saleh N. (2014). Antibiotic dispensation by Lebanese pharmacists: A comparison of higher and lower socio-economic levels. J. Infect. Public Health.

[65] Zgheib N.K., Sleiman F., Nasreddine L., Nasrallah M., Nakhoul N., Isma’eEl H., Tamim H. (2018). Short Telomere Length is Associated with Aging, Central Obesity, Poor Sleep and Hypertension in Lebanese Individuals. Aging Dis..

[66] Darwish H., Farran N., Assaad S., Chaaya M. (2018). Cognitive reserve factors in a developing country: Education and occupational attainment lower the risk of dementia in a sample of Lebanese older adults. Front. Aging Neurosci..

[67] Rousseau R., Garcia-Zorita C., Sanz-Casado E. (2023). Publications during COVID-19 times: An unexpected overall increase. J. Inf..

[68] Akinlotan O., Jalo A. (2024). “I Am Actually Scared of Everyone”: Older Adults’ Experiences of Social Isolation during COVID-19: A Qualitative Systematic Review. Covid.

[69] Maral P., Punetha D. (2022). Older adult life in COVID-19 pandemic: Focus on social isolation, loneliness, and minimization of risks. Ind. Psychiatry J..

[70] Abou Ltaif S.F., Mihai-Yiannaki S., Thrassou A. (2024). Lebanon’s Economic Development Risk: Global Factors and Local Realities of the Shadow Economy Amid Financial Crisis. Risks.

[71] Nassar M., Abdallah W., Najib B., Khalil K., Atallah D. (2023). Weakening of the Lebanese health sector. East. Mediterr. Health J..

[72] UNESCWA (2026). Conflict and Its Shockwaves: Older Persons Amid War and Displacement in Lebanon.

[73] UNESCWA (2021). Launch of the National Strategy for Older Persons in Lebanon 2020–2030.

[74] Issa Nauffal D. (2009). Do educational outcomes in Lebanese universities differ based on the academic model?. Educ. Bus. Soc. Contemp. Middle East. Issues.

[75] Auanassova A. (2023). International collaboration: Benefits and challenges. Cent. Asian J. Med. Hypotheses Ethics.

[76] Khan H.T.A., Addo K.M., Findlay H. (2024). Public Health Challenges and Responses to the Growing Ageing Populations. Public Health Chall..

[77] Zhang Y., Gu Z., Xu Y., He M., Gerber B.S., Wang Z., Liu F., Peng C. (2024). Global scientific trends in healthy aging in the early 21st century: A data-driven scientometric and visualized analysis. Heliyon.

[78] Waddell C., Van Doorn G., Power G., Statham D. (2024). From Successful Ageing to Ageing Well: A Narrative Review. Gerontologist.

[79] Abi Chahine M., Kienzler H. (2022). Ageism, an invisible social determinant of health for older Syrian refugees in Lebanon: A service providers’ perspective. Confl. Health.

[80] Jafari Bidgoli M., Gregg A., Stager C.G., Crowther M. (2026). Financial perceptions and subjective well-being among older adults. Front. Psychol..

[81] Kricheldorff C., Aner K., Himmelsbach I., Thiesemann R. (2015). Grundlagen der Sozialen Gerontologie. Z. Gerontol. Geriatr..

[82] Savino M., De Luca L., Nocentini A., Menesini E. (2026). Intergenerational interventions and their impact on active aging: A systematic review. Int. Psychogeriatr..

[83] Coelho A., Lopes M., Barata M., Sousa S., Goes M., Bia F., Dias A., João A., Lusquinhos L., Oliveira H. (2023). Biopsychosocial Factors That Influence the Purpose in Life among Working Adults and Retirees. Int. J. Environ. Res. Public Health.

[84] Kanning M., Schlicht W. (2008). A bio-psycho-social model of successful aging as shown through the variable “physical activity”. Eur. Rev. Aging Phys. Act..

[85] Fotteler M.L., Mühlbauer V., Brefka S., Mayer S., Kohn B., Holl F., Swoboda W., Gaugisch P., Risch B., Denkinger M. (2022). The Effectiveness of Assistive Technologies for Older Adults and the Influence of Frailty: Systematic Literature Review of Randomized Controlled Trials. JMIR Aging.

[86] Kisa A., Kisa S. (2026). Community-based interventions to support aging in place and functional independence in older adults: A systematic review of randomized controlled trials. Front. Public Health.

[87] Moody E., McDougall H., Paus Jenssen E., McArthur C., Affoo R., Weeks L.E., Macdonald M., Mojbafan A., Langman E. (2026). The effectiveness of alternatives to residential care for older people with on-going health and social care needs: A systematic review. BMC Geriatr..

[88] Buma L.E., Vluggen S., Mouchaers I., Zwakhalen S.M.G., Metzelthin S.F. (2025). Feasibility of a Reablement Programme in Community Care in the Netherlands; A Qualitative Study. Int. J. Integr. Care.

[89] Straughan P., Tan Y.W., Tiew Z., Zheng Z., Ngu R., Hiah W.T. (2025). Ageing in Place—The Key to Receiving a Superaged Society. Populations.

[90] Rhodes A., McNichols C.C. (2025). Aging in Place and Healthcare Equity: Using Community Partnerships to Influence Health Outcomes. Healthcare.

[91] Zhao Y., Chung P.-K. (2017). Neighborhood environment walkability and health-related quality of life among older adults in Hong Kong. Arch. Gerontol. Geriatr..

[92] Alagood J., Senn W.D., Prybutok G. (2025). Hybrid Analysis of Videoconference Technology Use by Aging-in-Place Organizations to Promote Social Engagement for Older Adults: A Scoping Review with Latent Topic Modeling. Healthcare.

[93] Jaana M., Lévesque Ryan M., Tamim H., Riachy E., Paré G. (2025). Perspectives of Older Adults on Assistive Technology: Qualitative Study. JMIR Hum. Factors.

[94] Wardlow L., Roberts C., Archbald-Pannone L., On Behalf of The Collaborative for Telehealth and Aging (2023). Perceptions and Uses of Telehealth in the Care of Older Adults. Telemed. e-Health.

[95] Leff B., Ritchie C.S., Rising K.L., Cannon K., Wardlow L. (2025). Addressing barriers to equitable telehealth for older adults. Front. Med..

[96] Chen P.-Y., Ho W.-C., Lo C., Yeh T.-P. (2021). Predicting Ego Integrity Using Prior Ego Development Stages for Older Adults in the Community. Int. J. Environ. Res. Public Health.

[97] Rose G.A., Bruni P.T., Wingood M., Kallmi S., Finer E., Bamonti P.M. (2025). A Systematic Review of the Effects of Therapeutic Exercise With Psychological Interventions on Disability and Personal Outcomes in Older Adults. Arch. Rehabil. Res. Clin. Transl..

[98] Yu D.S., Li P.W., Lin R.S.-Y., Kee F., Chiu A., Wu W. (2023). Effects of non-pharmacological interventions on loneliness among community-dwelling older adults: A systematic review, network meta-analysis, and meta-regression. Int. J. Nurs. Stud..

[99] Markle-Reid M., Ploeg J., Fraser K.D., Fisher K.A., Bartholomew A., Griffith L.E., Miklavcic J., Gafni A., Thabane L., Upshur R. (2018). Community Program Improves Quality of Life and Self-Management in Older Adults with Diabetes Mellitus and Comorbidity. J. Am. Geriatr. Soc..

[100] Park K., Lee S., Yang J., Song T., Hong G.-R.S. (2019). A systematic review and meta-analysis on the effect of reminiscence therapy for people with dementia. Int. Psychogeriatr..

[101] Skosireva A., Gobessi L., Eskes G., Cassidy K.-L. (2025). Effectiveness of enhanced group cognitive behaviour therapy for older adults (CBT-OA) with depression and anxiety: A replication study. Int. Psychogeriatr..

[102] Yang H., Zhong Q., Han B., Pu Y., He R., Huang K., Jiao Y., Han R., Kong Q., Jia Y. (2025). Effects of reminiscence therapy for loneliness in older adults: A systematic review and meta-analysis. Age Ageing.

[103] Yu T., Sy A., Wang A., Kamra M., Yu P., Pasumarthi T., Tangri N., Morgan R.L. (2026). Social support interventions for life satisfaction of older adults in long-term care: A systematic review and meta-analysis. Aging Health Res..

[104] Barakat Z., Sacre H., Khatib S., Hajj A., Malham C.B., Haddad C., Akel M., Zeenny R.M., Abbas L.A., Barakat M. (2025). Examining burden among caregivers of community-dwelling older adults in Lebanon. Sci. Rep..

[105] Ruiz-Grao M.C., Álvarez-Bueno C., Garrido-Miguel M., Berlanga-Macias C., Gonzalez-Molinero M., Rodríguez-Martín B. (2024). Multidisciplinary home-based interventions in adverse events and quality of life among frail older people: A systematic review and meta-analysis. Heliyon.

[106] Dehi M., Mohammadi F. (2020). Social Participation of Older Adults: A Concept Analysis. Int. J. Community Based Nurs. Midwifery.

[107] Donovan N.J., Blazer D. (2020). Social Isolation and Loneliness in Older Adults: Review and Commentary of a National Academies Report. Am. J. Geriatr. Psychiatry.

[108] Kang H., Kim H. (2022). Ageism and Psychological Well-Being Among Older Adults: A Systematic Review. Gerontol. Geriatr. Med..

[109] Younes R.S., Ziade C., Moukarzel C., Abboud Mzawak M. (2026). Loneliness, Isolation, and Well-Being Among Older Adults in Arab Countries: A Bibliometric Analysis and Systematic Review. Soc. Sci..

[110] Manor S. (2019). Ageing, ageism, and lost honor: Narratives of Arab elders in Israel. Int. J. Ageing Later Life.

[111] Massey E., Smith J., Roberts B. (2017). Health needs of older populations affected by humanitarian crises in low- and middle-income countries: A systematic review. Confl. Health.

[112] Sihombing N., Rahmadi M.A., Nasution H., Mawar L. (2025). Impact of Displacement on Mental Health Among Elderly War Victims in Syam. J. Ris. Ilmu Kesehat. Umum Dan Farm. JRIKUF.

[113] Boulle P. (2026). War in Lebanon: The hidden health crisis beyond the frontlines. BMJ.

[114] Hoh J.W.T., Lu S., Yin Y., Feng Q., Dupre M.E., Gu D., Gu D., Dupre M.E. (2021). Environmental Gerontology. Encyclopedia of Gerontology and Population Aging.

[115] Prina M., Khan N., Akhter Khan S., Caicedo J.C., Peycheva A., Seo V., Xue S., Sadana R. (2024). Climate change and healthy ageing: An assessment of the impact of climate hazards on older people. J. Glob. Health.

[116] El Chamieh C., El Haddad C., El Khatib K., Jalkh E., Al Karaki V., Zeineddine J., Assaf A., Harb T., Sanayeh E.B. (2024). River water pollution in Lebanon: The country’s most underestimated public health challenge. East. Mediterr. Health J..

[117] Caristia S., Viola E., Kalemi T., Servetti D., Poy S., Faggiano F. (2026). Policies and strategies on active and healthy ageing: A scoping review of the recommendations of European and international agencies. Front. Public Health.

[118] Khomsi K., Bouzghiba H., Mendyl A., Al-Delaimy A.K., Dahri A., Saad-Hussein A., Balaw G., El Marouani I., Sekmoudi I., Adarbaz M. (2024). Bridging research-policy gaps: An integrated approach. Environ. Epidemiol..

[119] UNESCWA (2026). Abuse of Older Persons in Lebanon: Silence and Policy Gaps.

[120] Jiang S., Guan R., Guo C., Wei C. (2024). Prevalence of Motoric Cognitive Risk Syndrome Among Community-Dwelling Older Adults: A Systematic Review and Meta-Analysis. J. Gerontol. Nurs..

[121] Teraz K., Šlosar L., Paravlić A.H., De Bruin E.D., Marusic U. (2022). Impact of Motor-Cognitive Interventions on Selected Gait and Balance Outcomes in Older Adults: A Systematic Review and Meta-Analysis of Randomized Controlled Trials. Front. Psychol..

[122] Pratali L., Mastorci F., Vitiello N., Sironi A., Gastaldelli A., Gemignani A. (2014). Motor Activity in Aging: An Integrated Approach for Better Quality of Life. Int. Sch. Res. Not..

[123] Gade Haanes G. (2024). Comprehensive Strategies for Enhancing Quality of Life in Older Adults with Sensory Impairments. HSOA J. Gerontol. Geriatr. Med..

[124] Tseng Y.-C., Liu S.H.-Y., Lou M.-F., Huang G.-S. (2018). Quality of life in older adults with sensory impairments: A systematic review. Qual. Life Res..

[125] Alessi C.A., Martin J.L. (2018). Sleep in Older Adults: Challenges and Opportunities. Sleep Med. Clin..

[126] Alhajaji R., Jahrami H., Pandi-Perumal S.R., BaHammam A.S. (2026). Sleep health in the older adults: Architecture, circadian changes, and common sleep disorders. Ageing Res. Rev..

[127] Li Z., Zhong T., Meng X. (2025). A meta-analysis study evaluating the effects of sleep quality on mental health among the adult population. BMC Public Health.

[128] Pizzol D., Demurtas J., Celotto S., Maggi S., Smith L., Angiolelli G., Trott M., Yang L., Veronese N. (2021). Urinary incontinence and quality of life: A systematic review and meta-analysis. Aging Clin. Exp. Res..

[129] Singh D.K.A., Murukesu R.R., Shahar S. (2019). Prevalence of urinary incontinence and its association with declined cognitive and physical function among community dwelling older adults: A review. Malays. J. Public Health Med..

[130] Bramer W.M., Rethlefsen M.L., Kleijnen J., Franco O.H. (2017). Optimal database combinations for literature searches in systematic reviews: A prospective exploratory study. Syst. Rev..

[131] Salvador-Oliván J.A., Marco-Cuenca G., Arquero-Avilés R. (2019). Errors in search strategies used in systematic reviews and their effects on information retrieval. J. Med. Libr. Assoc..

[132] Musharrafieh U., Dergham J., Daou C., Tamim H., Houry R., Bizri A.R. (2019). Influenza vaccine and cardiac protection: A study from a tertiary care center. Hum. Vaccines Immunother..

[133] Dalle I.A., Yassine A., Awada A., Chakhachiro Z., Mahfouz R., Moukalled N., El Cheikh J., Bazarbachi A. (2026). Cladribine, low-dose cytarabine, and venetoclax in newly diagnosed and relapsed/refractory acute myeloid leukemia: A global perspective. Curr. Res. Transl. Med..

[134] Hadi C.E., Saliba R., Maalouly G., Riachy M., Sleilaty G. (2025). Unsupervized clustering reveals a tri-phenotype model of hospitalized COVID-19 patients: Beirut cohort study and literature synthesis. Digit. Health.

[135] Doumat G., Daher D., Itani M., Abdouni L., Asmar K.E., Assaf G. (2023). The effect of polypharmacy on healthcare services utilization in older adults with comorbidities: A retrospective cohort study. BMC Prim. Care.

[136] El Halabi I., El Husseini Z., Haibe Y., Charafeddine M., Mukherji D., Temraz S., Bulbul M., Khauli R., Nasr R., Wazzan W. (2020). Cystectomy vs. bladder preservation after neoadjuvant chemotherapy in muscle-invasive bladder cancer: A tertiary medical center experience. Cancer Treat. Res. Commun..

[137] Koubaissi S.A., Daou M.A.Z., Mohamad R., Husari A. (2021). Increased incidence of massive hemorrhage at uncommon sites after initiation of systemic anticoagulation in critically ill patients with coronavirus disease 2019 (COVID-19) infection. J. Thromb. Thrombolysis.

[138] Jallad M.A., Naoufal R., Irani J., Azar E. (2015). Extended Spectrum Beta-Lactamase Carriage State among Elderly Nursing Home Residents in Beirut. Sci. World J..

[139] Mahfouz R., Zeidane R.A., Diab T., Tarhini A., Sbaity E., Kazarian H., El Zibaoui Y., Naji N.S., Barake M., Assi H.I. (2025). Real-World Data: Implementation and outcomes of Next-Generation sequencing in the MENA region. Diagnostics.

[140] Hosn M.A., Nasser Z., Elias E., Medawar W., Daouk M., Hoballah J., Haddad F. (2017). Switching temporary hemodialysis catheters to long-term catheters: Exchange versus de-novo placement, any difference in line infection?. Clin. Nephrol..

[141] Dakik H.A., Sbaity E., Msheik A., Kaspar C., Eldirani M., Chehab O., Hassan O.A., Mailhac A., Makki M., Tamim H. (2020). AUB-HAS2 Cardiovascular Risk Index: Performance in surgical subpopulations and comparison to the revised cardiac risk index. J. Am. Heart Assoc..

[142] Abboud G., Maalouly J., Tawk A., Aouad D., Ayoubi R., Najm T., El-Hajj G., El Rassi G., Nehme A. (2021). Intertrochanteric fractures treated by diaphyseal support arthroplasty with hook plate vs cerclage wires only: A retrospective cohort study. Ann. Med. Surg..

[143] Sayigh R. (2015). Yusif Sayigh: Arab Economist and Palestinian Patriot: A Fractured Life Story.

