# Supplementary material for: Toward Holistic Aging in Lebanon: A Mapping of Gerontology and Geriatrics Research Trends, Gaps, and Future Directions
Source: Healthcare (Basel). 2026 Sep 4;14(17):2857. doi: 10.3390/healthcare14172857 (PMC13565685; doi:10.3390/healthcare14172857)
Supplement: Supplementary file 1 [file healthcare-14-02857-s001.zip › healthcare-4523186-supplementary.pdf]

Brief Sensitivity Analysis

To evaluate the retrieval accuracy of our geographic parameters, a search sensitivity analysis was conducted comparing our primary affiliation-based filtering strategy against a topic keyword-based geographic search strategy. This methodological step was performed to empirically assess whether relying on institutional affiliations rather than geographic text keywords introduced any potential field truncation or omitted relevant literature on aging in Lebanon.

Supplementary Table S1. Brief Sentiment Analysis

**Search**	**Full search query**	**Number of items**	**Description**	**Outcome**	**Search date**
Search A (Original Strategy)	(TITLE-ABS-KEY (gerontology) OR TITLE-ABS-KEY (geriatric) OR TITLE-ABS-KEY (geriatrics) OR TITLE-ABS-KEY (older adults) OR TITLE-ABS-KEY (older people) OR TITLE-ABS-KEY (elderly) OR TITLE-ABS-KEY (elder) OR TITLE-ABS-KEY (nursing home) OR TITLE-ABS-KEY (retirement)) AND PUBYEAR > 2014 AND PUBYEAR < 2027 AND (LIMIT-TO (LANGUAGE, "English") OR LIMIT-TO (LANGUAGE, "French") OR LIMIT-TO (LANGUAGE, "Arabic")) AND (LIMIT-TO (AFFILCOUNTRY, "Lebanon"))	2,073	Primary search strategy using core aging/geriatric terms combined with the database country affiliation filter (AFFILCOUNTRY).	Formed the core dataset analyzed in the bibliometric review. Demonstrated high recall of local empirical studies.	10 May 2026
Search B (Keyword Strategy)	(TITLE-ABS-KEY (gerontology) OR TITLE-ABS-KEY (geriatric) OR TITLE-ABS-KEY (geriatrics) OR TITLE-ABS-KEY (older adults) OR TITLE-ABS-KEY (older people) OR TITLE-ABS-KEY (elderly) OR TITLE-ABS-KEY (elder) OR TITLE-ABS-KEY (nursing home) OR TITLE-ABS-KEY (retirement)) AND PUBYEAR > 2014 AND PUBYEAR < 2027 AND (LIMIT-TO (LANGUAGE, "English") OR LIMIT-TO (LANGUAGE, "French") OR LIMIT-TO (LANGUAGE, "Arabic")) AND (TITLE-ABS-KEY (leban* OR beirut))	619	Alternative geographic search strategy replacing country affiliation filters with topic keywords for Lebanon and Beirut (TITLE-ABS-KEY).	Retrieved a considerably smaller number of items, suggesting a portion of potentially useful publications were not recalled.	23 August 2026

Search C (Non-Overlapping Set)	[Search B Syntax] AND NOT (LIMIT-TO (AFFILCOUNTRY, "Lebanon"))	13	Comparative set identifying records captured by the geographic keyword string (Search B) but omitted by the affiliation filter (Search A).	Title and abstract screening of all 13 records yielded 0 eligible studies, suggesting minimal loss of relevant literature by using the primary strategy.	23 August 2026

Note. Search A was conducted during initial dataset extraction on May 10, 2026, whereas Searches B and C for the sensitivity analysis were executed during the first round of peer review (August 2026).

Supplementary Table S2. Screening Results for Non-Overlapping Records (Search C)

Authors	Title	Decision
Musharrafieh U., Dergham J., Daou C., Tamim H., Houry R., Bizri A.R. [132]	Influenza vaccine and cardiac protection: a study from a tertiary care center	Did not meet inclusion criteria
Abou Dalle I., Yassine A., Awada A., Chakhachiro Z., Mahfouz R., Moukalled N., El Cheikh J., Bazarbachi A. [133]	Cladribine, low-dose cytarabine, and venetoclax in newly diagnosed and relapsed/refractory acute myeloid leukemia: A global perspective	Did not meet inclusion criteria
El Hadi C., Saliba R., Maalouly G., Riachy M., Sleilaty G. [134]	Unsupervized clustering reveals a tri-phenotype model of hospitalized COVID-19 patients: Beirut cohort study and literature synthesis	Did not meet inclusion criteria
Doumat G., Daher D., Itani M., Abdouni L., El Asmar K., Assaf G. [135]	The effect of polypharmacy on healthcare services utilization in older adults with comorbidities: a retrospective cohort study	Did not meet inclusion criteria
Halabi I.E., Husseini Z.E., Haibe Y., Charafeddine M., Mukherji D., Temraz S., Bulbul M., Khauli R., Nasr R., Wazzan W., Hajj A.E., Geara F., Shamseddine A. [136]	Cystectomy vs. bladder preservation after neoadjuvant chemotherapy in muscle-invasive bladder cancer: A tertiary medical center experience	Did not meet inclusion criteria
Koubaissi S.A., Daou M.A.Z., Mohamad R., Husari A. [137]	Increased incidence of massive hemorrhage at uncommon sites after initiation of systemic anticoagulation in critically ill patients with coronavirus disease 2019 (COVID-19) infection	Did not meet inclusion criteria
Jallad M.A., Naoufal R., Irani J., Azar E. [138]	Extended spectrum beta-lactamase carriage state among elderly nursing home residents in beirut	Did not meet inclusion criteria
Mahfouz R., Abou Zeidane R., Diab T., Tarhini A., Sbaity E., Kazarian H., El Zibaoui Y., Naji N.S., Barake M., Assi H.I. [139]	Real-World Data: Implementation and Outcomes of Next-Generation Sequencing in the MENA Region	Did not meet inclusion criteria
Hosn M.A., Nasser Z., Elias E., Medawar W., Daouk M., Hoballah J., Haddad F. [140]	Switching temporary hemodialysis catheters to long-term catheters: Exchange versus de-novo placement, any difference in line infection?	Did not meet inclusion criteria
Dakik H.A., Sbaity E., Msheik A., Kaspar C., Eldirani M., Chehab O., Hassan O.A., Mailhac A., Makki M., Tamim H. [141]	Aub-has2 cardiovascular risk index: Performance in surgical subpopulations and comparison to the revised cardiac risk index	Did not meet inclusion criteria

Abboud G., Maalouly J., Tawk A., Aouad D., Ayoubi R., Najm T., El-Hajj G., El Rassi G., Nehme A. [142]	Intertrochanteric fractures treated by diaphyseal support arthroplasty with hook plate vs cerclage wires only: A retrospective cohort study	Did not meet inclusion criteria
Jallad M.A., Naoufal R., Irani J., Azar E. [143138]	Erratum: Corrigendum to "extended Spectrum Beta-Lactamase Carriage State among Elderly Nursing Home Residents in Beirut"(Scientific World Journal (2015) :(1) 10.1155/2015/750527)	Did not meet inclusion criteria
Sayigh R. [144143]	YUSIF SAYIGH: Arab Economist, Palestinian Patriot: A Fractured Life Story	Did not meet inclusion criteria
